# Supplementation of Protein at Breakfast Rather Than at Dinner and Lunch Is Effective on Skeletal Muscle Mass in Older Adults

**DOI:** 10.3389/fnut.2021.797004

**Published:** 2021-12-21

**Authors:** Hyeon-Ki Kim, Hanako Chijiki, Mayuko Fukazawa, Jin Okubo, Mamiho Ozaki, Takuya Nanba, Seiichirou Higashi, Miho Shioyama, Masaki Takahashi, Takashi Nakaoka, Shigenobu Shibata

**Affiliations:** ^1^Faculty of Science and Engineering, Waseda University, Shinjuku City, Japan; ^2^Graduate School of Advanced Science and Engineering, Waseda University, Shinjuku City, Japan; ^3^Meiji Co., Ltd, Koto City, Japan; ^4^Institute for Liberal Arts, Tokyo Institute of Technology, Meguro City, Japan; ^5^Saitama Cancer Center, Saitama, Japan

**Keywords:** protein intake timing, protein intake at breakfast, physical function, muscle function, skeletal muscle mass, older adults

## Abstract

**Background:** The effects of different intake patterns of meal protein on muscle mass have not been clarified. We cross-sectionally and longitudinally examined the effect of different timing of protein intake on sarcopenia-related factors in older adults.

**Methods:** This cross-sectional study 1 included 219 (male, *n* = 69, female, *n* = 150) elderly subjects aged ≥65 years. Subjects who consumed more protein at breakfast than at dinner were grouped into the morning group (MG, *n* = 76; male, *n* = 26; female, *n* = 50), and those who consumed more protein at dinner than at breakfast were grouped into the evening group (EG, *n* = 143; male, *n* = 43; female, *n* = 100). In cross-sectional study 2-1 (female, *n* = 125), the subjects were classified into four groups according to the number of meals with sufficient protein intake. In cross-sectional studies 2-2 (female, *n* = 125) and 2-3 (female, *n* = 27), the subjects were classified into eight groups and three groups according to whether they had consumed sufficient protein at three meals; sarcopenia-related factors were compared. The intervention study was a placebo-controlled, double-blind, randomized controlled trial that included 40 elderly women with low daily breakfast protein intake. The subjects were divided into four groups: morning protein and placebo intake groups and evening protein and placebo intake groups. Each group consumed the test food (containing 10 g milk protein) or placebo in the morning or evening for 12 weeks. Blood indices and physical function were assessed before and after the intervention.

**Results:** Comparing all subjects, MG showed significantly higher handgrip strength than did EG (*P* < 0.05). The higher ratio of morning protein intake relative to the total protein intake, the better the muscle mass (*r* = 0.452, *P* < 0.05) and handgrip strength (*r* = 0.383, *P* < 0.05). The intervention study showed an increase in muscle mass with the intake of milk protein in the morning rather than in the evening (*P* < 0.05).

**Conclusions:** Protein intake at breakfast might have relatively stronger effects on skeletal muscle mass than at lunch and dinner.

## Introduction

As we age, skeletal muscle mass decreases, causing a decline in muscle strength and physical function (1), a condition known as sarcopenia, which increases the risk of impaired physical independence in the elderly (2). Therefore, maintaining or increasing muscle mass could prevent sarcopenia.

Muscle hypertrophy occurs when the rate of muscle protein synthesis (MPS) exceeds that of muscle protein breakdown (MPB), while muscle atrophy occurs when the rate of MPS falls below that of MPB. Since MPS is stimulated by dietary protein, protein intake is important for maintaining and increasing muscle mass (3). In addition, the sensitivity of MPS to stimulation by amino acid intake is reduced in the elderly compared to the young. Therefore, elderly people need to consume more protein (4).

Recently, it has been suggested that factors such as the quality and distribution of daily protein intake are more relevant to muscle synthesis than the amount of daily protein intake. In fact, previous studies have shown that consuming enough protein in all three meals is more effective in maintaining and improving muscle mass, strength, and physical function (5). Furthermore, it has been confirmed that genes involved in muscle synthesis and degradation have circadian rhythms, and muscle synthesis may have a diurnal rhythm (6).

Many studies regarding protein intake and sarcopenia-related factors have been conducted on healthy subjects, and few studies have been conducted on obese people, and those requiring support (5). Insulin resistance is common in individuals with obesity. Since insulin can promote MPS, high insulin resistance may inhibit muscle synthesis (7). Protein intake in the morning has been reported to improve insulin sensitivity (8) which could promote MPS. The term “those who require support” is equivalent to frailty; it has been reported that many people with frailty have a bias toward one meal a day with protein (9). Few studies have examined the distribution of protein intake, and none have focused on the effects of differences in the timing of protein intake on sarcopenia-related factors (10, 11). In the present study, we examined the effects of morning or evening protein intake on sarcopenia-related factors in elderly people who were healthy, obese, or requiring support in a cross-sectional study (cross-sectional study 1). Since a protein intake of 0.4 g/kg body weight (BW) per meal is known to be the cut-off value that maximizes the stimulation of MPS (4), we defined a meal with a protein intake exceeding 0.4 g/kg BW as a “sufficient protein intake meal” and examined the effects of different protein intake patterns (i.e., different timing of the three meals with sufficient protein intake) on sarcopenia-related factors in elderly women (cross-sectional study 2). Finally, we aimed to examine the effect of 10 g milk protein supplementation in the morning or evening on sarcopenia-related factors in elderly women with inadequate morning protein intake, which is common in Japan (intervention study).

## Methods

### Cross-Sectional Study

#### Participants

Two hundred and nineteen adults aged 65 years or older were included in the study (72.5 ± 0.4 years, mean age ± standard error [SE]). The subjects were healthy, obese, or required support; the study was conducted from August 2017 to February 2019 in Tokyo and Hokkaido (Japan). Subjects who required support in this study were those able to live on their own but needing partial assistance in their daily activities and have been certified by the nursing care insurance system in Japan. Some of the data used in this study were collected earlier for a previous study on the effects of morning or evening protein intake on the physical functions of the elderly (*N* = 60) (12).

Subjects without regular exercise habits were included in this study. Regular exercise habit was defined as continuous physical activity for at least 30 min per session, at least three times per week, as defined by a previous study (13). Subjects who were habitual smokers or had smoked within the past three years, and those who had suffered from cardiovascular disease in the past and/or had a pacemaker were excluded from this study. All subjects completed a questionnaire on dietary intake, lifestyle habits, health, and medication status prior to study enrollment.

All subjects were fully briefed on the outline and safety of the study, and written consent to participate was obtained. The study protocol conformed to the Helsinki Declaration and was approved by the ethics committee for humans at Waseda University (approval numbers: 2017-231, 2018-031, 2018-137).

#### Study Protocol

**Figure 1:**
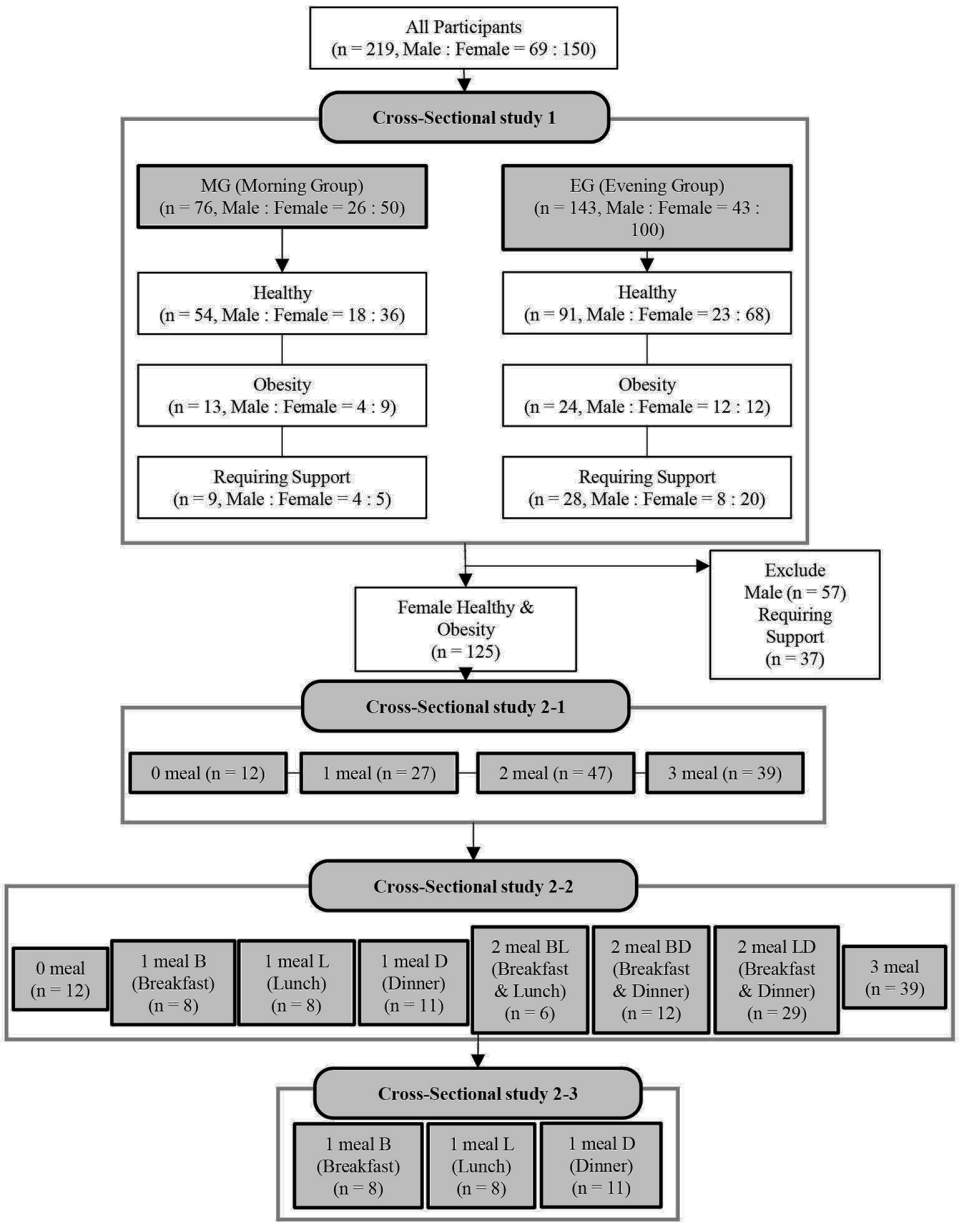
Flow diagram showing the design of the cross-sectional study. B, breakfast; L, lunch; D, dinner.

In cross-sectional study 1, we examined the effect of different timings of protein intake on sarcopenia-related factors in all subjects. All subjects were divided into two groups: those who consumed more protein at breakfast than at dinner were grouped into the morning group (MG) and those who consumed more protein at dinner than at breakfast were grouped into the evening group (EG) (Figure 1).

In cross-sectional study 2, 125 healthy or obese female subjects (71.1 ± 0.4 years, mean age ± SE) were examined for the effects of different patterns of protein intake (depending on whether or not they had sufficient protein intake at breakfast, lunch, and dinner) on sarcopenia-related factors. In the cross-sectional study 2-1, the subjects were classified into four groups according to the number of meals with sufficient protein intake (Figure 1): 0 meals, 1 meal, 2 meals, and 3 meals. In the cross-sectional study 2-2, the protein intake patterns were classified into eight patterns according to whether the protein intake during the three meals (breakfast, lunch, and dinner) was sufficient (Figure 1; Supplementary Figure 1). In other words, those who had zero meals with sufficient protein intake were classified into the 0 meal group, and those who had sufficient protein intake at only one meal were classified into the 1 meal B (breakfast), 1 meal L (lunch), and 1 meal D (Dinner) groups. Those with sufficient protein intake at only two meals (breakfast and lunch) were classified into the 2 meals BL group, those with sufficient protein intake at only two meals (breakfast and dinner) into the 2 meals BD group, those with sufficient protein intake at only two meals (lunch and dinner) into the 2 meals LD group, and those with all three meals into the 3 meals group. In the cross-sectional study 2-3, we compared three groups (1 meal B, 1 meal L, and 1 meal D) in which protein intake was sufficient at only one meal (Figure 1).

### Measurements

#### Anthropometry

BW was measured to the nearest 0.1 kg using a digital balance (Inbody 230, Inbody Inc., Tokyo, Japan) and height was measured to the nearest 0.1 cm using a wall-mounted stadiometer (YS-OA, As One Corp., Japan). Body mass index (BMI) was calculated as weight in kilograms divided by the square of height in meters.

#### Muscle Mass, Muscle Strength, and Functional Test

Muscle mass was measured by direct segmental multi-frequency (20 kHz to 100 kHz) bioimpedance analysis (InBody230, InBody Co., Ltd., Tokyo, Japan). The skeletal muscle index (SMI) was calculated as the skeletal muscle mass (kg) divided by the square of height (m). Muscle strength was measured using a digital hand dynamometer (T.K.K.5401, Takei Scientific Instruments Co., Ltd., Niigata, Japan). The handgrip strength of the dominant hand was measured twice while standing, and the mean of these measurements was used for analysis. Gait speed test was also evaluated. The subjects walked a 5 m straight course at a normal speed. The walking time for the mid 3m of the course was measured using a digital gait speed measuring instrument (YW, Yagami Co., Ltd., Tokyo, Japan), and the gait speed (m/s) was calculated by dividing the distance in meters by the time in seconds.

#### Physical Activity Assessment

All participants were asked to wear a triaxial accelerometer (Active style Pro HJA-750C; Omron Corp., Kyoto, Japan) for a week. They always wore the accelerometer each day from morning until night, except during shower and bedtimes. In this study, we selected 4 weekdays and one holiday in which the wearer wore the device for at least 10 h (600 min) and averaged the data to calculate the daily physical activity. We used moderate-to-vigorous physical activity (MVPA) and the number of steps required for evaluation. All minute recordings that were ≥3 METs (metabolic equivalents) were classified as MVPA (14).

#### Chronotype Assessment

To determine the chronotype of the subjects (morningness to eveningness), a lifestyle survey was conducted using the Morningness-Eveningness Questionnaire (MEQ) (15). The scores ranged from 16 to 86 points. The participants were divided into the following three chronotype groups: morningness (score 59–86), intermediate (score 42–58), or eveningness (score 16–41).

#### Dietary Assessment

The Food Frequency Questionnaire (FFQ) was used to assess the dietary and nutritional intake of the subjects. It consists of questions on 29 food groups and 10 cooking methods. Most FFQs for Japanese are highly effective in estimating nutrients (16). Average daily energy intake is depicted as kilocalories per day (kcal/day). The total dietary fiber quantity was described as grams per day (g/day). In addition, protein-related items such as animal products (meat, eggs, milk, and fish), protein-rich vegetables (beans and soybeans), and dairy products were assessed for protein intake at each meal (breakfast, lunch, and dinner) and recorded.

### Intervention Study

#### Participants

Forty healthy elderly women aged ≥65 years (69.5 ± 0.7 years, mean age ± SE) who consented to participate in the present study and whose daily breakfast protein intake did not meet 0.4 g/kg BW were included in the intervention study. This study included elderly women without regular exercise habits and the inclusion criteria were as follows: (1) no antioxidant, anti-obesity, or anti-diabetes supplements use; (2) no medical diagnosis of diabetes, dyslipidemia, or sleep apnea syndrome; (3) no hypertension (systolic blood pressure: >140 mmHg, diastolic blood pressure: >90 mmHg). Therefore, it was only intended for subjects who had been confirmed to have no medication use, disease history, or smoking habits at the initial recruitment stage.

This study was conducted in Tokyo (Japan) from August 2017 to February 2019. The participants were those who had no special lifestyle changes during the intervention period and did not have a regular exercise habit.

This study was approved by the Ethics Committee of Waseda University (approval no. 2017-231) and was conducted in accordance with the guidelines established in the Declaration of Helsinki. The human trial of the present study was registered at https://upload.umin.ac.jp/cgi-open-bin/ctr_e/ctr_view.cgi?recptno=R000032737 as UMIN000028612.

#### Study Design

**Figure 2:**
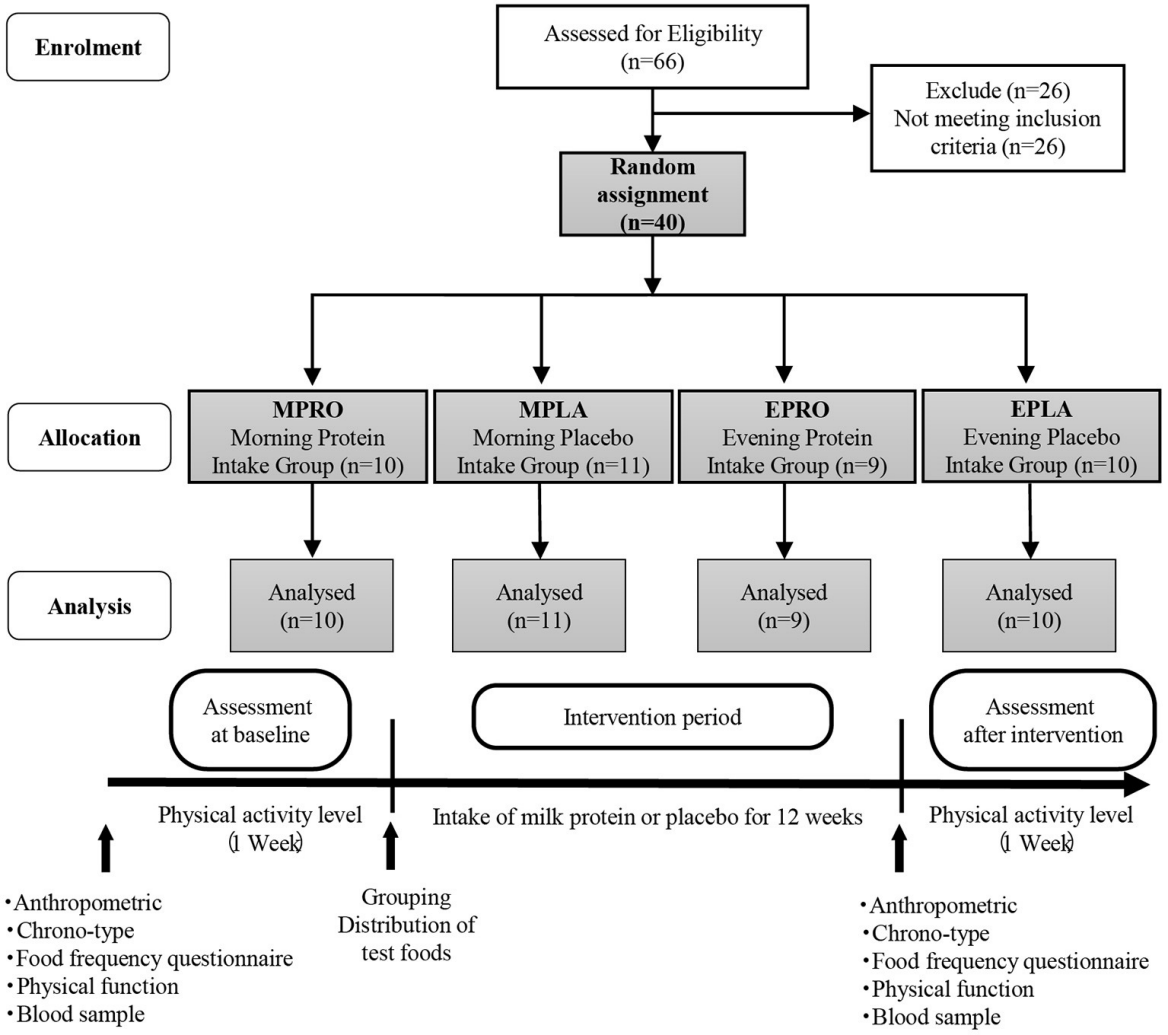
Flow diagram showing the design of the intervention study and protocol.

A placebo-controlled, double-blind, randomized controlled trial was conducted. Forty subjects were randomly assigned to one of the following four groups: the morning protein group (MPRO, *n* = 10), morning placebo group (MPLA, *n* = 11), evening protein group (EPRO, *n* = 9), and evening placebo group (EPLA, *n* = 10) (Figure 2). The random assignment of subjects was performed by someone other than the researchers. It was done by using a sequence generated by combining the RAND and RANK functions of Microsoft Excel. During the intervention period of 12 weeks, subjects in the MPRO and MPLA groups consumed milk protein or placebo in the morning (6:00–10:00 a.m.), and subjects in the EPRO and EPLA groups consumed milk protein or placebo in the evening (6:00–10:00 p.m.). The test food was consumed at home. We asked the participants to keep a diary to record their intake of the test foods and we checked their intake rates. The intake rate of the test foods was above 80% for all participants.

#### Protein Content

Meiji Co., Ltd provided both the test food (containing milk protein) and the control food (placebo) used in the present study. We asked Meiji Co., Ltd, the organization that provided the test foods, to have the participants and the person in charge of the experiment pack the test foods in such a way that they could not be identified, and then mail them to each subject. Disclosure regarding the test foods was made after the experiment was completed. As summarized in Supplementary Table 1, milk protein contained 10 g/meal total protein, while placebo contained 0 g total protein. Each test food was also matched and adjusted based on appearance and flavor such that they could not be distinguished. The participants dissolved the milk protein or placebo in 150 ml or more of water and consumed it at the specified time. Milk protein and placebo were available in two flavors (plain and matcha) and were selected according to the subject's preference.

#### Anthropometry, Physical Activity Level, Chronotype, Energy Intake, Muscle Mass, and Physical Function

Anthropometry, physical activity levels, chronotype, and energy intake were assessed before and after the intervention using the same methods as in the cross-sectional studies. In addition to the items investigated in the cross-sectional study, the subjects participating in the intervention study underwent measurements of appendicular skeletal muscle mass and appendicular skeletal muscle index (ASMI), physical fitness tests (Balance test, Time Up and Go (TUG), and “Sit to Stand” Test), taken before and after the intervention.

ASMI was calculated as appendicular skeletal muscle mass (kg) divided by the square of height (m). A one-leg stand test was conducted to evaluate the balance ability of the participants. The participants were instructed to stand on one leg on a flat surface for as long as possible. The arms were held by the body with the eyes opened. If the participant was able to stand on one leg for more than 120 s, the measurement was stopped at 120 s. The measurement was performed twice, and a better record was used for the analysis. The sit-to-stand test was performed five times to assess lower extremity muscle strength. When measuring the time taken to change from a sitting to a standing position and vice versa, participants were instructed to stand up from sitting five times as quickly as possible without using their arms for support. The total duration was recorded in seconds. The TUG test was used to assess the mobility and balance of the subjects. The time (s) it took for the subject to stand up from a sitting position in a chair, walk to 3 m, and then return to the original chair position was measured.

#### Blood Sample

Venous blood samples were collected before and after the intervention. The participants were required to refrain from any strenuous exercise for at least a day before the collection of the blood sample and fast for at least 12 h overnight. The participants' fasting blood was collected at 10–11 am the following day. After collection, blood for serum analysis was allowed to stand for 30 min at room temperature, whereas blood for plasma analysis was centrifuged at 3,500 rpm for 10 min. After centrifugation, serum and plasma samples were extracted from the respective blood collection tubes and stored at −80°C until the day of the assay. Plasma insulin, glucose, and serum growth hormone (GH) were analyzed by Kotobiken Medical Laboratories, Inc. (Tokyo, Japan).

### Statistical Analysis

The Predictive Analytics Software for Windows (SPSS Japan Inc. Tokyo, Japan) was used for data analysis. The total sample size was calculated to be able to detect a medium effect. Total sample size of 128 (Cross-sectional study) and 48 (Intervention study) were required to have ~80% power to detect large effects at a significance level of 0.05 (G^*^Power, version 3.1.9.2, Universitat Kiel, Germany). The normal or non-normal distributions of the data were analyzed using the Shapiro–Wilk test. In cross-sectional study 1, the mean values between the MG and EG groups were compared, and data that were normative and equally distributed were evaluated using the unpaired *t*-test. Data that were not confirmed to be normative or equally distributed were evaluated using Mann-Whitney's U test. In cross-sectional studies 2 and 3, data with normality and equal variance were evaluated using one-way analysis of variance (ANOVA), while data without normality or equal variance were evaluated using the Kruskal-Wallis test. Pearson's product-moment correlation coefficient was used to examine the relationship between the rate of protein intake in each meal and physical function.

The intervention study used a two-way ANOVA to compare the rate of change in physical function before and after the intervention among the groups. When normality and equality of variance were not confirmed, the Wilcoxon's *t*-test was used for the corresponding data, while the Mann-Whitney U test was used for the non-corresponding data. Statistical significance was set at *P* < 0.05. Additionally, *P* < 0.1 was considered a statistically significant tendency.

## Results

### Cross-Sectional Study 1

#### Characteristics of Participants and Energy Intake

We compared the characteristics of all participants and within the different subject categories of the MG and EG. There was no significant difference in physical characteristics between the groups for all subjects, obese subjects, and those requiring support. However, age was significantly higher in healthy MG, especially in healthy women of the MG, compared to those in the EG (*P* < 0.05) (Supplementary Table 2).

There were no significant differences in energy intake and total protein intake between the groups (Supplementary Table 3).

#### Comparison of Sarcopenia-Related Factors

Comparing all subjects (*n* = 219; MG = 69, EG = 150), MG showed significantly higher handgrip strength than did EG (*P* < 0.05). Correcting handgrip strength for each subject's weight reduced the significance of the MG's higher handgrip strength (*P* = 0.054). In all healthy subjects (*n* = 145; MG = 54, EG = 91), SMI and handgrip strength of the MG were significantly higher than those of the EG (*P* < 0.05, respectively), and the MG tended to have higher muscle mass than the EG (*P* = 0.071). In contrast, there were no statistically significant differences between groups among all obese subjects (*n* = 37; MG = 13, EG = 24) and all those requiring assistance (*n* = 37; MG = 9, EG = 28).

Considering the influence of sex differences, we also examined the results separately for men and women. In all men (healthy, obese, and those requiring support), there were no statistically significant differences in sarcopenia-related factors between the groups. On the other hand, healthy female subjects (*n* = 104; MG = 36, EG = 68) showed significantly higher values or a trend toward higher values for SMI, handgrip strength, and handgrip strength (weight-corrected) in the MG than in the EG (*P* < 0.01, *P* < 0.05, *P* = 0.069). In addition, in women with obesity (*n* = 21; MG = 9, EG = 12), handgrip strength and handgrip strength (weight-corrected) were significantly higher in the MG than in the EG (*P* < 0.05, *P* < 0.001) (Table 1).

**Table 1:** Comparison of sarcopenia-related factors between MG and EG (cross-sectional study 1).

**All**	**All participants**		**Healthy (*n* = 145)**		**Obesity (*n* = 37)**		**Participants(*n* = 37)** **requiring support (*n* = 37)**	
	**MG (*n* = 76)**	**EG (*n* = 143)**	**MG (*n* = 54)**	**EG (*n* = 91)**	**MG (*n* = 13)**	**EG (*n* = 24)**	**MG (*n* = 9)**	**EG (*n* = 28)**
Muscle mass (kg)	21.35 ± 0.49	20.75 ± 0.35	21.13 ± 0.58	20.04 ± 0.38	22.72 ± 1.25	24.25 ± 0.96	20.71 ± 1.42	20.05 ± 0.78
Muscle mass (kg/BW)	0.38 ± 0.01	0.37 ± 0.00	0.39 ± 0.01	0.38 ± 0.00	0.34 ± 0.01	0.36 ± 0.01	0.35 ± 0.02	0.34 ± 0.01
SMI (kg/m^2^)	8.53 ± 0.12	8.39 ± 0.09	8.35 ± 0.12^#^	8.05 ± 0.09	9.08 ± 0.31	9.53 ± 0.19	8.84 ± 0.35	8.52 ± 0.21
Hand grip (kg)	25.88 ± 0.86^#^	24.10 ± 0.65	26.17 ± 1.03^#^	23.65 ± 0.73	27.03 ± 1.76	28.40 ± 1.79	22.46 ± 2.80	21.84 ± 1.52
Hand grip (kg/BW)	0.46 ± 0.01	0.42 ± 0.01	0.48 ± 0.02^#^	0.44 ± 0.01	0.41 ± 0.02	0.42 ± 0.02	0.38 ± 0.04	0.37 ± 0.02
Gait speed (m/s)	1.42 ± 0.03	1.35 ± 0.02	1.48 ± 0.03	1.44 ± 0.02	1.40 ± 0.05	1.39 ± 0.04	1.07 ± 0.07	1.03 ± 0.05
**Male**	**MG (*n* = 26)**	**EG (*n* = 43)**	**MG (*n* = 18)**	**EG (*n* = 23)**	**MG (*n* = 4)**	**EG (*n* = 12)**	**MG (*n* = 4)**	**EG (*n* = 8)**
Muscle mass (kg)	25.72 ± 0.66	25.76 ± 0.53	25.44 ± 0.83	25.06 ± 0.64	28.18 ± 1.29	27.87 ± 1.12	24.50 ± 1.47	24.61 ± 1.10
Muscle mass (kg/BW)	0.42 ± 0.01	0.40 ± 0.00	0.43 ± 0.01	0.42 ± 0.01	0.38 ± 0.01	0.39 ± 0.01	0.39 ± 0.01	0.39 ± 0.01
SMI (kg/m^2^)	9.34 ± 0.19	9.55 ± 0.13	9.10 ± 0.20	9.25 ± 0.13	10.26 ± 0.57	10.27 ± 0.20	9.52 ± 0.53	9.32 ± 0.41
Hand grip (kg)	32.92 ± 1.42	33.36 ± 0.91	33.65 ± 1.65	33.05 ± 1.18	34.58 ± 2.28	36.32 ± 1.19	27.96 ± 4.98	29.84 ± 2.65
Hand grip (kg/BW)	0.54 ± 0.03	0.52 ± 0.01	0.57 ± 0.03	0.55 ± 0.02	0.47 ± 0.02	0.51 ± 0.02	0.44 ± 0.07	0.47 ± 0.04
Gait speed (m/s)	1.41 ± 0.05	1.34 ± 0.04	1.46 ± 0.07	1.43 ± 0.05	1.36 ± 0.11	1.34 ± 0.06	1.22 ± 0.10	1.08 ± 0.09
**Female**	**MG (*n* = 50)**	**EG (*n* = 100)**	**MG (*n* = 36)**	**EG (*n* = 68)**	**MG (*n* = 9)**	**EG (*n* = 12)**	**MG (*n* = 5)**	**EG (*n* = 20)**
Muscle mass (kg)	19.08 ± 0.37	18.59 ± 0.21	18.97 ± 0.45	18.34 ± 0.21	20.3 ± 0.84	20.63 ± 0.46	17.68 ± 0.87	18.23 ± 0.66
Muscle mass (kg/BW)	0.36 ± 0.01	0.35 ± 0.00	0.37 ± 0.00	0.36 ± 0.00	0.33 ± 0.01	0.33 ± 0.01	0.32 ± 0.02	0.33 ± 0.01
SMI (kg/m^2^)	8.11 ± 0.10	7.90 ± 0.07	7.97 ± 0.12^**^	7.65 ± 0.06	8.56 ± 0.22	8.79 ± 0.10	8.30 ± 0.34	8.20 ± 0.20
Hand grip (kg)	22.21 ± 0.63^**^	20.11 ± 0.41	22.43 ± 0.76^*^	20.48 ± 0.45	23.67 ± 1.13^*^	20.49 ± 0.80	18.06 ± 1.55	18.64 ± 1.30
Hand grip (kg/BW)	0.42 ± 0.01^*^	0.38 ± 0.01	0.44 ± 0.01	0.41 ± 0.01	0.38 ± 0.02^**^	0.33 ± 0.01	0.34 ± 0.05	0.33 ± 0.02
Gait speed (m/s)	1.43 ± 0.03	1.36 ± 0.03	1.50 ± 0.03	1.44 ± 0.02	1.43 ± 0.05	1.43 ± 0.06	0.98 ± 0.07	1.01 ± 0.06

*Values are expressed as mean and standard errors*.

* *P < 0.05*,

** *P < 0.01 compared to EG (t-test)*.

# *P < 0.05 compared to EG (Mann-Whitney). MG, Morning Group; EG, Evening Group; BW, Body Weight; SMI, Skeletal Muscle Index*.

### Effect of Number of Melas With Adequate Protein Intake on Sarcopenia-Related Factors (Cross-Sectional Study 2-1)

**Figure 3:**
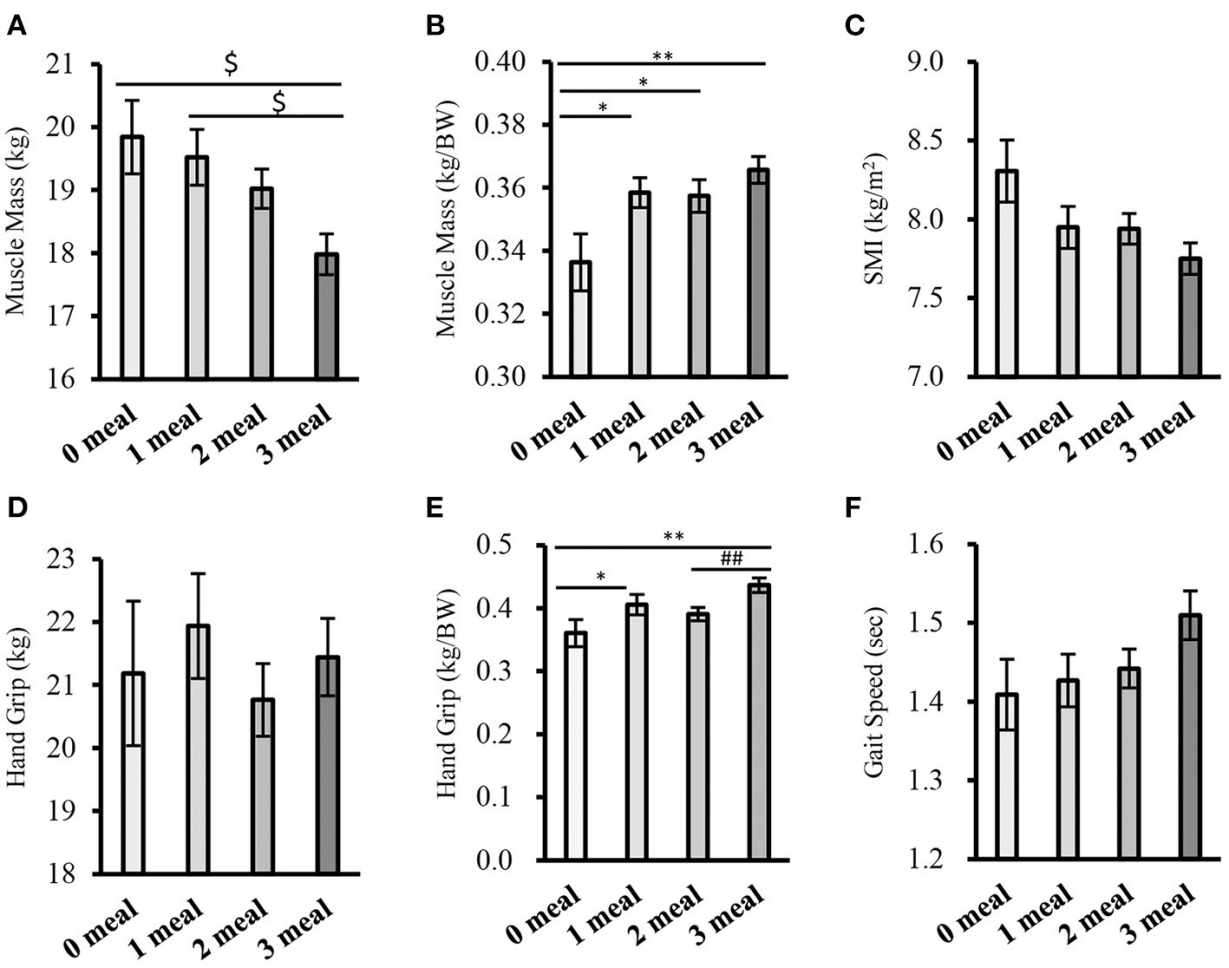
Comparison of sarcopenia-related factors by the number of meals meeting 0.4g/kg BW (cross-sectional study 2-1). **(A)** Muscle mass, **(B)** Muscle mass (corrected for body weight), **(C)** SMI, **(D)** Handgrip strength, **(E)** Handgrip strength (weight-corrected), **(F)** Gait speed. Values are expressed as mean and standard error. ^$^*P* < 0.05 compared to 3 meals (one-way ANOVA), *P < 0.05, **P < 0.01 compared to 0 meal (Mann-Whitney), ^##^*P* < 0.01, compared to 3 meals (Mann-Whitney), BW, Body Weight; SMI, Skeletal Muscle Index.

Subjects with fewer meals of adequate protein intake had significantly higher values for BW, BMI, percentage fat, and fat mass (Supplementary Table 4). The results of the comparison of sarcopenia-related factors according to the number of meals meeting 0.4 g/kg BW are shown in Figures 3A–F. The higher the number of meals with adequate protein intake, the significantly higher the muscle mass (weight-corrected) (0 meal group vs. 1 meal group, 2 meals group, 3 meals group: *P* < 0.05, *P* < 0.05, *P* < 0.01, respectively) and handgrip strength (weight-corrected) (0 meal group vs. 1 meal group, 3 meals group: *P* < 0.05, *P* < 0.01, respectively; 2 meals group vs. 3 meals group; *P* < 0.01) (Figures 3B,E). In terms of gait speed, those who had more meals with sufficient protein intake tended to walk faster (0 meal group vs. 3 meals group; *P* = 0.053, 1 meal group vs. 3 meals group; *P* = 0.076) (Figure 3F).

### Effects of Eight Different Protein Intake Patterns on Sarcopenia-Related Factors (Cross-Sectional Study 2-2)

Protein intake patterns were classified into eight groups, according to whether the protein intake during the three meals was sufficient or not, and the subjects belonging to each intake pattern were divided into eight groups for comparison (Supplementary Figure 1).

**Figure 4:**
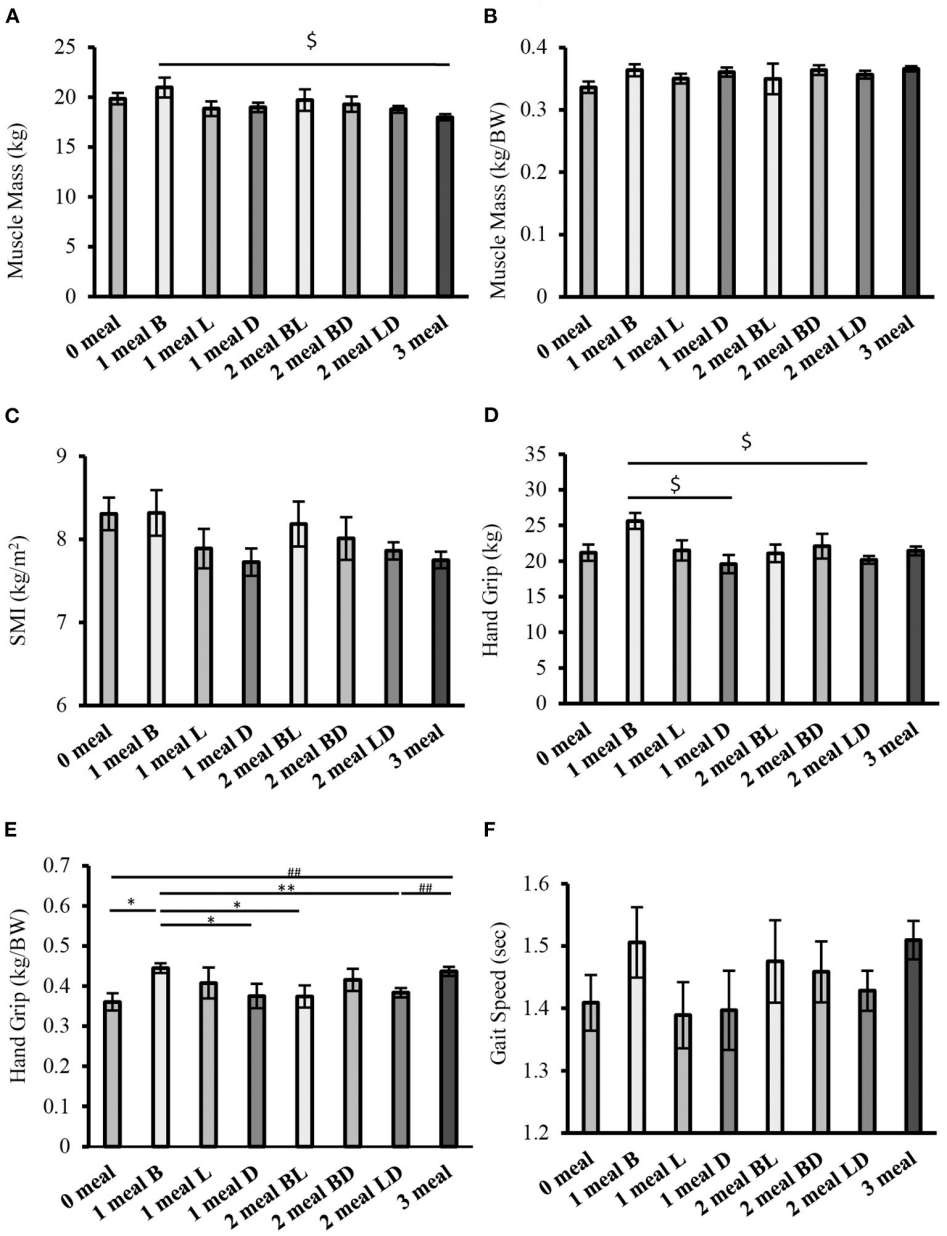
Comparison of sarcopenia-related factors by protein intake pattern (cross-sectional study 2-2). **(A)** Muscle mass, **(B)** Muscle mass (weight-corrected), **(C)** SMI, **(D)** Handgrip strength, **(E)** Handgrip strength (weight-corrected), **(F)** Gait speed. Values are expressed as mean and standard error. ^$^*P* < 0.05 compared to 1 meal B (one-way ANOVA), *P < 0.05, **P < 0.01 compared to 1 meal B (Mann-Whitney), ^##^*P* < 0.01, compared to 3 meals (Mann-Whitney), BW, Body Weight; SMI, Skeletal Muscle Index; B, Breakfast, L, Lunch; D, Dinner; BL, Breakfast and Lunch; BD, Breakfast and Dinner; LD, Lunch and Dinner.

The results of the comparison of sarcopenia-related factors by protein intake patterns are shown in Figures 4A–F. There were no significant differences in muscle mass (weight-corrected) or gait speed between the groups (Figures 4B,F). However, handgrip strength (weight-corrected) was significantly higher in the 1 meal B group than in the 0 meal, 1 meal D, 2 meals BL, and 2 meals LD groups (one meal group vs. 0 meal, one meal D, and two meals BL groups; *P* < 0.05, respectively; 1 meal group vs. 2 meals LD group; *P* < 0.01). In addition, handgrip strength (weight-corrected) was significantly higher or tended to be higher in the 3 meals group than in the 0 meal group, 1 meal D group, 2 meals BL group, or 2 meals LD group (3 meals group vs. 0 meal group, 2 meals LD group; *P* < 0.01, respectively; 3 meals group vs. 1 meal D group, 2 meals BL group; *P* = 0.059, *P* = 0.053) (Figure 4E).

Handgrip strength (weight-corrected) was lower in the group that consumed sufficient protein at both breakfast and lunch than in the group that consumed sufficient protein at only one meal in the morning. If we consider the importance of protein intake in the morning, we can assume that the comparison of the two groups would yield similar results. However, the results of this study differ from our hypotheses.

### Comparison of Three Groups With Sufficient Protein Intake at One Meal (Cross-Sectional Study 2-3)

To examine the effects of protein intake at breakfast, lunch, and dinner in more detail, we compared sarcopenia-related factors in three groups that had sufficient protein intake at only one meal. There were no significant differences in physical characteristics or energy intake between the groups (Supplementary Table 5).

**Figure 5:**
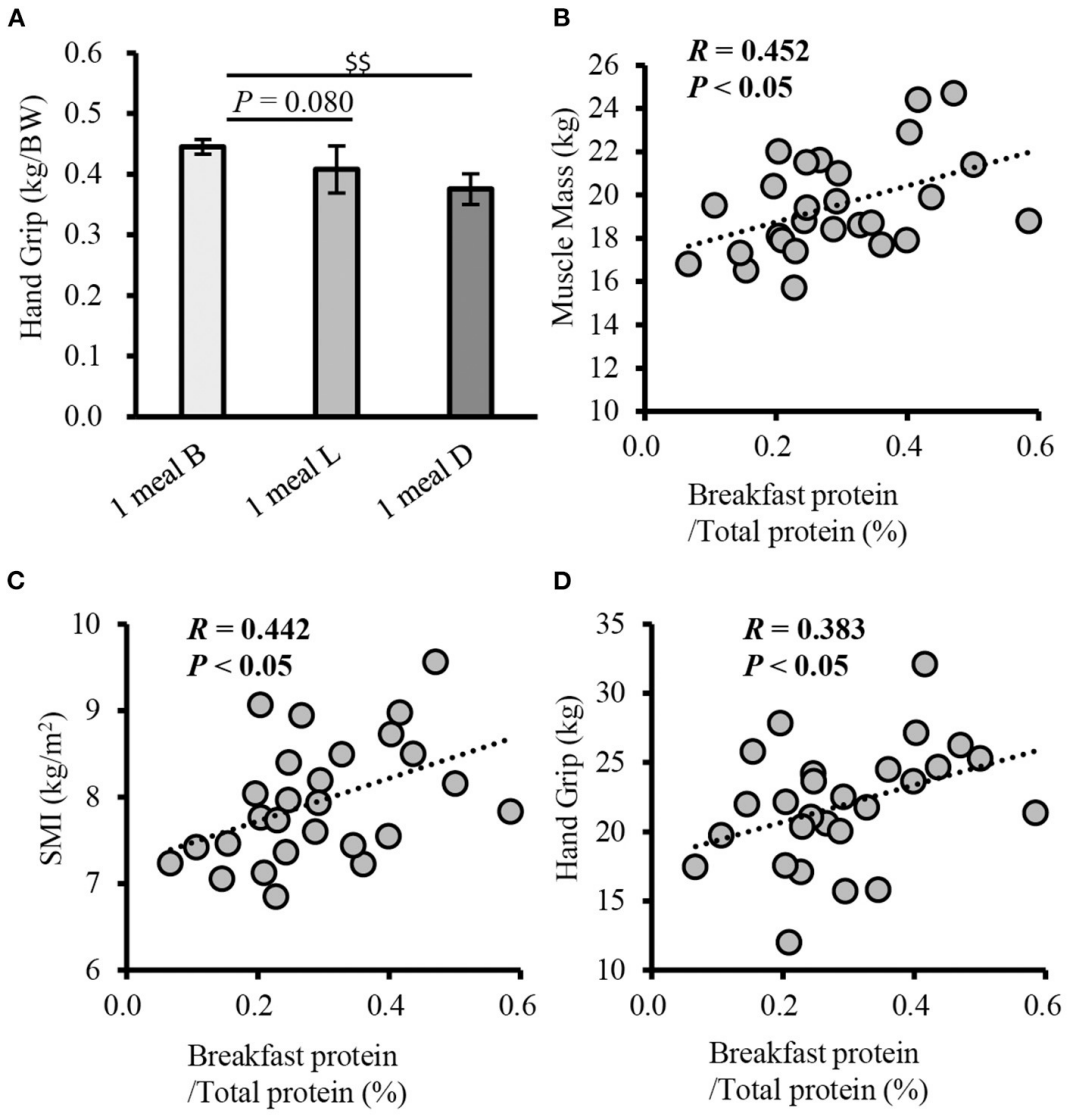
Comparison of handgrip strength between patterns with only one meal of adequate protein intake, and correlation of the rate of breakfast protein intake with physical function (cross-sectional study 2-3). **(A)** Comparison of grip strength between patterns with only one meal of adequate protein intake, Correlation between muscle mass **(B)**, SMI **(C)**, handgrip strength **(D)**, and the rate of breakfast protein intake. Values are expressed as mean and standard error. ^$$^*P* < 0.01, compared to 1 meal D (one-way ANOVA). Pearson's product-moment correlation coefficient. SMI, skeletal muscle index; B, breakfast; L, lunch; D, inner.

In terms of sarcopenia-related factors, handgrip strength (weight-corrected) was higher in the 1 meal B group than in the 1 meal L and 1 meal D groups (*P* = 0.080 and *P* < 0.001, respectively) (Figure 5A). Additionally, handgrip strength was higher in the 1 meal B group than in the 1 meal D group (*P* < 0.001) (Table 2). Furthermore, the correlation between each item and the ratio of breakfast protein intake to total protein intake was examined. Muscle mass, SMI, and handgrip strength were positively correlated with the proportion of breakfast protein intake (muscle mass, *r* = 0.452; SMI, *r* = 0.442; handgrip strength, *r* = 0.383; *P* < 0.05, respectively) (Figures 5B–D). However, there was no statistically significant association between each item and the ratio of lunch and dinner protein intake to total protein intake.

**Table 2:** Comparison of sarcopenia-related factors between patterns of adequate protein intake with only one meal (Cross-sectional study 2-3).

	**1 meal B**	**1 meal L**	**1 meal D**
	**(*n* = 8)**	**(*n* = 8)**	**(*n* = 11)**
Muscle Mass (kg)	20.96 ± 1.00	18.84 ± 0.74	18.96 ± 0.51
Muscle mass (kg/BW)	8.32 ± 0.27	7.89 ± 0.24	7.72 ± 0.18
SMI (kg/m^2^)	0.36 ± 0.01	0.35 ± 0.01	0.36 ± 0.01
Hand grip (kg)	25.62 ± 1.11^$$^	21.50 ± 1.42	19.57 ± 1.10
Hand grip (kg/BW)	0.45 ± 0.01	0.41 ± 0.04	0.38 ± 0.03
Gait speed (m/s)	1.51 ± 0.06	1.39 ±0.05	1.40 ± 0.06

*Values are expressed as mean and standard errors*.

$$ *P < 0.01 compared to 1 meal D (One-Way ANOVA). BW, Body Weight; SMI, Skeletal Muscle Index; B, Breakfast; L, Lunch; D, Dinner*.

### Effect of Milk Protein Supplementation in The Morning or Evening on Sarcopenia-Related Factors in Elderly Women With Routinely Inadequate Morning Protein Intake (Intervention Study)

There were no significant differences between characteristics of participants, and pre-intervention energy intake in each group (Supplementary Tables 6, 7).

**Figure 6:**
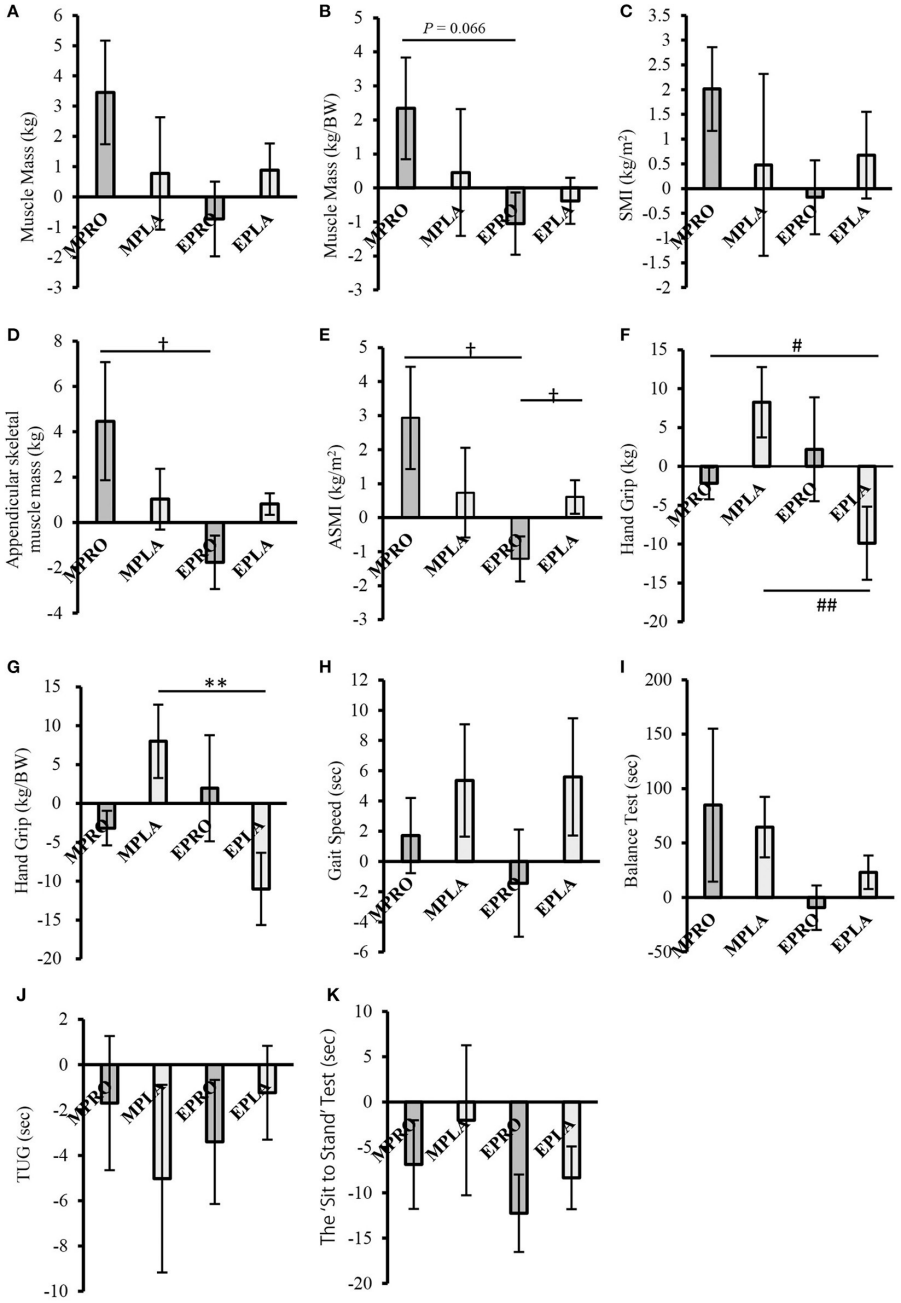
Comparison of the rate of change of sarcopenia-related factors by intervention (intervention study) **(A)** Muscle mass, **(B)** Muscle mass (weight-corrected), **(C)** SMI, **(D)** Appendicular skeletal muscle mass, **(E)** ASMI, **(F)** Handgrip strength, **(G)** Handgrip strength (weight-corrected), **(H)** Gait speed, **(I)** One-leg stand test, **(J)** TUG, **(K)** The “Sit to Stand” Test. Values are expressed as the mean ± standard error. ^†^*P* < 0.05, compared to EPRO (Mann-Whitney). ^#^*P* < 0.05, ^##^*P* < 0.01, compared to EPLA (Mann-Whitney). **P < 0.01 compared to EPRO (Two-Way ANOVA). MPRO, morning protein intake group; MPLA, morning placebo intake group; EPRO, evening protein intake group; EPLA, evening placebo intake group; BW, body weight; SMI, skeletal muscle index; ASMI, appendicular skeletal muscle index; TUG, time up and go.

The results of the comparison of the rate of change of sarcopenia-related factors by intervention are shown in Figures 6A–K. In muscle mass (weight-corrected), ASMM, and ASMI, the rate of change in the MPRO group significantly exceeded or tended to exceed the rate of change in the EPRO group (muscle mass [weight-corrected]; *P* = 0.066, ASMM, and ASMI; *P* < 0.05, respectively) (Figures 6B,D,E). In addition, muscle mass, SMI, and ASMI tended to increase in the MPRO group compared to the pre-intervention (muscle mass (pre: 18.62 ± 0.66 kg, post: 19.22 ± 0.64 kg; *P* = 0.059), SMI (pre: 7.78 ± 0.22 kg/m^2^, post: 7.94 ± 0.22 kg/m2; *P* = 0.057), ASMI (pre: 5.69 ± 0.17 kg/m^2^, post: 5.85 ± 0.16 kg/m^2^; *P* = 0.061). In the EPRO group, ASMI showed a decreasing trend after the intervention compared with before the intervention (pre: 5.51 ± 0.07 kg/m^2^, post: 5.45 ± 0.10 kg/m^2^; *P* = 0.084).

In contrast, the rate of change in the EPLA group was significantly lower than that in the MPLA group in handgrip strength and handgrip strength (weight-corrected) (*P* < 0.01, respectively) (Figures 6F,G). In addition, in the within-group comparison, handgrip strength and handgrip strength (weight-corrected) decreased in the EPLA group after the intervention compared to before the intervention (handgrip strength (pre: 21.15 ± 1.25 kg, post: 19.03 ± 1.33 kg; *P* = 0.059), handgrip strength (weight-corrected) (pre: 0.41 ± 0.03 kg/BW, post: 0.36 ± 0.02 kg/BW; *P* < 0.05).

## Discussions

The present study was a cross-sectional and interventional investigation of the effects of different protein intake patterns on sarcopenia-related factors. The main results showed that adequate protein intake in the morning is important for maintaining muscle mass and strength, with the effect being more pronounced in women. In the intervention study, only morning milk protein intake over 12 weeks resulted in increased muscle mass. These results suggest that morning protein intake is also effective in improving muscle mass and strength.

### Relationship Between Pattern and Timing of Protein Intake and Sarcopenia-Related Factors

Sarcopenia-related factors, such as muscle mass, grip strength, and physical function, may be influenced by the MEQ scores and physical activity. Morningness has been shown to have higher daily physical activity than eveningness (17) and increased physical activity may result in higher muscle mass (18). However, in the present study, sarcopenia-related factors in healthy and obese subjects were not associated with the MEQ score or physical activity. Therefore, the higher muscle mass and strength in the MG are due to protein intake, rather than the effects of chronotype or physical activity level.

However, the higher muscle mass and strength in the MG may not be explained solely by the higher protein intake in the morning than in the evening. It has been reported that an even intake of protein at all three meals, rather than a bias toward the evening meal, is associated with higher muscle mass and strength (10, 19). In the present study, the MG was more likely to consume equal amounts of protein at breakfast, lunch, and dinner, and consequently had better values for sarcopenia-related factors. In fact, when we compared the protein intake of healthy elderly people (*n* = 145; MG: *n* = 54, EG: *n* = 91) divided by their BW (Supplementary Table 3), we found that the protein intake of MG exceeded 0.4 g/kg BW at three meals, while that of EG was below 0.4 g/kg BW in the morning. Therefore, to examine the effects of the different timing of protein intake on sarcopenia-related factors in more detail, we included the pattern of evening protein intake below 0.4 g/kg BW. Therefore, in cross-sectional study 2, we examined different patterns of dietary protein intake above 0.4 g/kg BW/meal.

In cross-sectional study 2-1, we examined how differences in the number of meals with more than 0.4 g/kg BW of protein affect sarcopenia-related factors. The results showed that the greater the number of meals with sufficient protein intake, the higher the muscle mass (weight-corrected) and grip strength (weight-corrected), and the faster the walking speed. These results are in agreement with those of previous studies (20). Since protein intake of 0.4 g/kg BW or more stimulates MPS, the more often protein is consumed above 0.4 g/kg BW, the more often MPS is stimulated. Therefore, the rate of MPS may have exceeded the rate of MPB more frequently in those who consumed sufficient protein, resulting in higher values of muscle mass and strength.

In cross-sectional study 2-2, we examined the effects of eight different patterns of protein intake timing, for the three meals, on sarcopenia-related factors. In cross-sectional study 2-3, we examined the effects of different timings of breakfast, lunch, and dinner on sarcopenia-related factors in people who consume enough protein in only one meal. The results showed that adequate protein intake at only one breakfast meal was more important for maintaining muscle strength than at only one lunch meal or one dinner meal. Furthermore, it was suggested that the effect of sufficient protein intake at only one breakfast meal on muscle function was equivalent to that of sufficient protein intake at all three meals.

It has been reported that 3.4% of genes expressed in the skeletal muscle exhibit circadian rhythms (684 genes are found in fast-twitch muscle and 1,359 in slow-twitch muscle) (21, 22). The detailed mechanism by which morning protein intake results in higher muscle mass and strength is unknown. However, previous studies have shown that protein synthesis in the skeletal muscle of mice is high during the early active phase (corresponding to the morning in humans), and expression of *Atrogin-1* and *MuRF-1*, genes involved in muscle degradation, is high from the late active phase to the early inactive phase (corresponding to nighttime in humans) (21, 23). These findings suggest that dietary proteins may be more available for muscle synthesis in the morning and less available in the evening. In fact, our recent study in rodents showed that mice consuming more protein at breakfast gained more muscle mass than mice that consumed more protein at dinner or consumed equal amounts in the morning and evening (12). Therefore, it is thought that a diet with enough protein in the morning could stimulate MPS to the maximum extent for muscle synthesis utilization because the amount of protein ingested was commensurate with the variation in muscle synthesis utilization of dietary protein. Thus, muscle mass and strength were maintained or increased and showed high values. In addition, when protein was consumed evenly in all three meals, the amount of protein at dinner was excessive, but the MPS for muscle synthesis utilization could be stimulated to the maximum extent, so that muscle mass and strength were maintained or increased to the same extent as when enough protein was consumed in the morning. Therefore, morning protein intake is more effective than evening protein intake in maintaining or increasing muscle mass and strength, and the effect is the same as when sufficient protein is consumed at all three meals.

### Differences in Protein Intake Balance and Sarcopenia-Related Factors Among Gender

This cross-sectional study showed that high protein intake in the morning maintained higher muscle mass and strength in healthy and obese elderly women. However, the same results were not obtained for men as for women. There are two possible reasons for this finding.

First, it is possible that women are more dependent on dietary protein for muscle synthesis than men. In a previous study, it was shown that the rate of muscle mass loss was lower with higher daily protein intake in elderly women, but there was no association between daily protein intake and muscle mass in elderly men (24). Furthermore, it has been reported that protein intake is associated with maintenance of grip strength and physical performance in women (25). It has also been reported that women have a higher MPS rate and a higher rate of myofibrillar synthesis of dietary protein than men, suggesting that women are more dependent on dietary protein for muscle synthesis than men (26). Therefore, it is possible that the muscle mass and strength of the elderly women in the MG were higher than those of the EG because they were more susceptible to the effects of differences in timing (differences in the efficiency of protein intake). In addition, testosterone secretion related to MPS is 10 times higher in men than in women, and muscle mass and strength are known to be higher in men than in women at all ages, despite the decrease in testosterone secretion with aging (27, 28). Therefore, men may have been more influenced by other factors, such as testosterone, in muscle synthesis than women, and may have been less affected by the timing of protein intake.

Second, the diurnal variation in muscle function-related genes may differ between men and women. Sex differences in the diurnal variation of muscle function-related genes have not yet been clarified. However, in the present study, the effect of the different timing of protein intake on muscle mass and strength was determined only in females. Therefore, there may be sex differences in the diurnal variation in muscle function-related genes. In other words, there may be a small diurnal variation in males and a large diurnal variation in females. In contrast, it has been reported that the expression of *MyoD*, a gene involved in muscle differentiation, has a diurnal rhythm controlled by clock genes and is greatly affected by diet (29, 30). In the present study, it is unclear how the difference in the timing of protein intake between morning and evening affected the expression of *MyoD*, but it is possible that morning protein intake in elderly women suppressed the decrease in muscle regeneration, which was weakened by the decrease in female hormone secretion via *MyoD*. However, it is possible that the morning protein intake in elderly women suppressed the decrease in muscle regeneration capacity, which was weakened by decreased female hormone secretion through *MyoD*.

### Differences in Protein Intake Balance and Sarcopenia-Related Factors in Obese People and Those Requiring Support

In cross-sectional study 1, we examined the association between different timing of protein intake and sarcopenia-related factors in obese people and people requiring support as well as healthy people. In obese subjects, as well as in healthy subjects, it was shown that women who consumed more protein in the morning had higher muscle strength. It was also shown that muscle strength was lower when the percentage of protein intake in the evening was high. This may be because the morning protein intake improved insulin resistance, which is worsened in obese people (8), and suppresses the decrease in MPS velocity.

On the other hand, among those requiring support, there was no difference in sarcopenia-related factors among subjects of both sexes who showed a balance of protein intake in the morning or evening. However, it was shown that muscle mass and strength were higher in subjects who needed support in the morning type. In the subjects requiring support in this study, we were not able to examine the effect of physical activity on muscle mass and strength because we were not able to sufficiently measure physical activity meters. However, it has been shown that the amount of daily physical activity is higher in morning-type people (17), and it is possible that the muscle mass and strength of the people requiring support showed higher values because the amount of physical activity was higher in morning-type people. Since people who require support have some difficulties in their daily activities, their physical functions may be lower than those of healthy or obese people. Therefore, it may be more important to increase the amount of physical activity than the balance of protein intake to maintain muscle mass, strength, and physical function.

### Different Timing of Milk Protein Intake and Sarcopenia-Related Factors

In this intervention study, the intake of milk protein in the morning rather than in the evening increased appendicular skeletal muscle mass and ASMI. Two factors may have contributed to this result.

The first factor is the increase in the number of meals with adequate protein intake. It is known that dietary protein has a dose-dependent effect on MPS stimulation (31). Therefore, supplementation of milk protein at breakfast (MPRO group), when protein intake is inadequate, increases protein intake in the morning and enhances MPS stimulation in a dose-dependent manner. Therefore, in the MPRO group, the frequency of MPS stimulation during the day increased, and appendicular skeletal muscle mass hypertrophy was thought to have occurred. In contrast, when milk protein was supplemented in the evening (EPRO group), the frequency of MPS stimulation did not increase as much as in the MPRO group because the protein was not supplemented in the morning when protein intake was insufficient and MPS stimulation occurred only in the afternoon or evening. Furthermore, because protein MPS stimulation has a dose-dependent effect, there is a threshold for this effect (4, 32), it is possible that the MPS stimulation of protein at dinner was already at the threshold and the MPS stimulation of milk protein supplemented in the evening was ineffective in those who had already consumed sufficient protein at dinner.

Second, based on the results of cross-sectional studies 2-2 and 2-3, it is possible that muscle mass was increased by consuming protein at the time of the morning. As shown in the results of cross-sectional study 1, the additional intake of milk protein in the morning may have increased appendicular skeletal muscle mass because the amount of protein consumed was commensurate with the variation in muscle synthesis utilization of dietary protein, which efficiently stimulated the MPS. In contrast, in the group that consumed additional milk protein in the evening, the protein required for the muscle synthesis utilization of dietary protein remained insufficient in the morning, and the MPS could not be stimulated to the maximum extent for muscle synthesis utilization, resulting in MPB being triggered, and a decrease in appendicular skeletal muscle mass may have been observed.

There was a significant difference in handgrip strength between the placebo groups; those who consumed the placebo in the morning showed an increase in handgrip strength compared to those who consumed the placebo in the evening. The reason for this difference is not clear, but it may be due to the higher carbohydrate content of the control food (placebo) compared to the test food. In previous studies, breakfast carbohydrate intake was reported to improve exercise performance (33). Therefore, the group that received the placebo in the morning may have increased their morning carbohydrate intake, leading to improved exercise performance.

In contrast, there was no statistically significant difference between MPRO and EPRO. However, there was a slight increase in the rate of change of handgrip strength before and after the intervention only in EPRO. In general, sports performance, such as muscle strength and flexibility, is associated with diurnal variation in body temperature, which has been reported to be greatest in the evening (34). However, it was not possible to clarify the effect on muscle strength in the timing of protein intake in the intervention trials of this study. On the other hand, the cross-sectional study in the present study showed that MG had higher handgrip strength compared to EG. The duration of the intervention study in this study was 12 weeks, which may have been insufficient to examine the changes in muscle strength. Therefore, it is necessary to clarify the effect of the timing of protein intake on muscle strength by examining a longer period of time in the future.

### Timing of Intake of Different Milk Proteins and Blood Indices

In a 12-week intervention study, differences in the timing of milk protein intake did not affect blood indices (insulin, glucose, and growth hormone) (Supplementary Table 8). Insulin and growth hormones are known to promote MPS (7, 35). Insulin promotes MPS by activating the mammalian target of rapamycin (mTOR) signal transduction pathway (7). On the other hand, in the elderly, insulin resistance increases with age, and the ability to synthesize MPS is reduced (36). In addition, GH replacement therapy has been reported to significantly increase skeletal muscle mass in healthy elderly men (37).

The lack of changes in these blood indices in the present study suggests that the intake of milk protein at different intake timings did not affect these blood indices. In addition, the increase in appendicular skeletal muscle mass in the MPRO group did not improve insulin resistance or the hormonal environment. Therefore, it is possible that the 12-week period of milk protein intake was too short, and that a longer intervention should be conducted in the future. In addition, the blood samples in this intervention study were taken in the fasting state, and we did not compare the blood indices in the morning and evening after protein intake before and after the intervention. Considering that skeletal muscle protein synthesis in the elderly is resistant to the anabolic effects of insulin and that this is an important factor in the development of sarcopenia (36), it is necessary to examine the changes in blood indices after protein intake in more detail. Furthermore, metabolome analysis showed that most postprandial metabolites related to the glycolytic system, tricarboxylic acid cycle, and amino acids were elevated in the morning compared to the evening, indicating that postprandial metabolic responses are higher in the morning (38). Therefore, a more detailed study including metabolites may help clarify the differences in metabolic responses related to skeletal muscle protein synthesis in the different protein intake balance between morning and evening.

### Limitations of Study

This study had several limitations. The study was conducted on elderly subjects. However, elderly people are less sensitive to the stimulation of muscle protein synthesis by amino acids than younger people (39). Since leucine has been shown to significantly inhibit activation of the mTOR signaling pathway (40), differences in protein intake may have different effects on muscle function in young adults. Therefore, further studies on young adults are required.

Cross-sectional study 2-2 showed that handgrip strength (weight-corrected) was lower in the group that consumed protein only at two meals, in the morning and at lunch, than in the group that consumed enough protein only at one meal in the morning. If it is important to consume protein in the morning, it is expected that muscle strength would show similar values when the group consumed enough protein at only one meal and when the group consumed enough protein at two meals: breakfast and lunch. This may be due to the small number of participants, as there were only six participants who consumed enough protein in two meals: breakfast and lunch. Therefore, there is a need to increase the number of elderly people with inadequate protein intake patterns in the future for a more detailed study.

Previous studies have not reached a consensus on the differences between men and women in muscle protein synthesis pathways, such as mTOR activity, due to diet and exercise, and much remains unclear (41, 42). In addition, there is still insufficient data to define whether the protein requirements of elderly men and women differ. Therefore, further investigation on the differences between men and women, including the expression rhythms of muscle function-related genes, is needed.

Calcium was a component of the test food. Several studies have reported no association between calcium intake and muscle mass or function (43–45). However, in adults over 50 years of age, low blood calcium levels may lead to decreased muscle mass. Particularly, a low calcium intake has been shown to be a possible predictor of muscle loss in women (46). Therefore, the effect of calcium intake on muscle mass and function, in this study, is undeniable. However, since the calcium intake of each protein group was similar, it is likely that the biological effects were comparable. Furthermore, more detailed studies are needed in the future to clarify the effects of calcium intake on muscle mass and muscle function.

## Conclusion

In conclusion, the current study suggests that adequate protein intake in the morning may be effective in maintaining and increasing muscle mass and strength, and this effect is particularly pronounced in older women. Furthermore, the results demonstrated that morning milk protein supplementation may lead to increased muscle mass in older women with inadequate morning protein intake.

## Data Availability Statement

The original contributions presented in the study are included in the article/Supplementary Materials, further inquiries can be directed to the corresponding author/s.

## Ethics Statement

The studies involving human participants were reviewed and approved by Ethics Committee for Humans at Waseda University. The patients/participants provided their written informed consent to participate in this study.

## Author Contributions

H-KK, HC, and SS: conceptualization. HC and H-KK: data curation. H-KK and HC: formal analysis. SS, SH, and MS: funding acquisition. H-KK, HC, MF, JO, MO, and TN: investigation. H-KK, HC, and SS: methodology. H-KK and SS: project administration. SS and H-KK: resources. SS: supervision. H-KK and HC: visualization. H-KK, HC, MT, and SS: writing—original draft. TN, MT, and SS: writing—review and editing. All authors read and approved the final manuscript.

## Funding

This work was supported by the Japan Society for the Promotion of Science (KAKENHI Grant Number 19H01089 to SS), JSTMirai Program Grant Number JMPJM120D5, Japan, and by Meiji Co., Ltd. The funder was not involved in the study design, collection, analysis, interpretation of data, the writing of this article or the decision to submit it for publication.

## Conflict of Interest

SH and MS were employed by company Meiji Co., Ltd. The remaining authors declare that the research was conducted in the absence of any commercial or financial relationships that could be construed as a potential conflict of interest.

## Publisher's Note

All claims expressed in this article are solely those of the authors and do not necessarily represent those of their affiliated organizations, or those of the publisher, the editors and the reviewers. Any product that may be evaluated in this article, or claim that may be made by its manufacturer, is not guaranteed or endorsed by the publisher.
