# Supplementary material for: Supplementation of Protein at Breakfast Rather Than at Dinner and Lunch Is Effective on Skeletal Muscle Mass in Older Adults
Source: Front Nutr. 2021 Dec 21;8:797004. doi: 10.3389/fnut.2021.797004 (PMC8724572; doi:10.3389/fnut.2021.797004)
Supplement: Supplementary file 1 [file Data_Sheet_1.docx]

Supplementary Material

# Supplementary Data

Supplementary Material should be uploaded separately on submission. Please include any supplementary data, figures and/or tables. All supplementary files are deposited to FigShare for permanent storage and receive a DOI.

Supplementary material is not typeset so please ensure that all information is clearly presented, the appropriate caption is included in the file and not in the manuscript, and that the style conforms to the rest of the article. To avoid discrepancies between the published article and the supplementary material, please do not add the title, author list, affiliations or correspondence in the supplementary files.

# Supplementary Figures and Tables

For more information on Supplementary Material and for details on the different file types accepted, please see [here](http://home.frontiersin.org/about/author-guidelines#SupplementaryMaterial). Figures, tables, and images will be published under a Creative Commons CC-BY licence and permission must be obtained for use of copyrighted material from other sources (including re-published/adapted/modified/partial figures and images from the internet). It is the responsibility of the authors to acquire the licenses, to follow any citation instructions requested by third-party rights holders, and cover any supplementary charges.

## Supplementary Figures

**
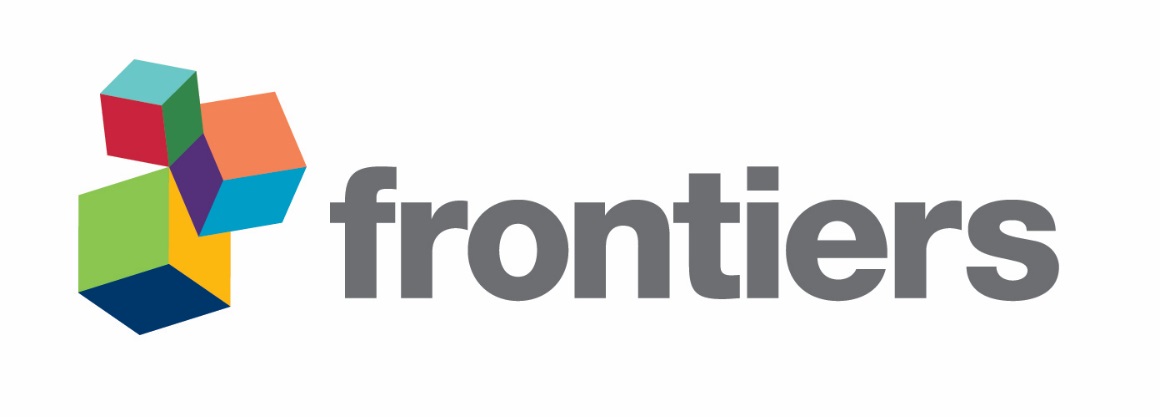
**

**Supplementary Figure 1.** The figure legends are required to have the same font as the main text, 12 point normal Times New Roman, single spaced. Please use a single paragraph for each legend and prepare the figures keeping in mind the PDF layout.

**Supplementary Table 1.** Test food and placebo contents.

|  | **Milk Protein (plain)** | **Milk Protein (matcha)** | **Placebo (plain)** | **Placebo (matcha)** |
| --- | --- | --- | --- | --- |
| **Energy (kcal)** | 47.1 | 47.8 | 47.2 | 47.2 |
| **Protein (g)** | 10.0 | 10.0 | 0 | 0 |
| **Fat (g)** | 0.2 | 0.2 | 0 | 0 |
| **Total carbohydrate (g)** | 1.2 | 1.3 | 11.8 | 11.8 |
| **Sodium chloride equivalent (g)** | 0.04 | 0.04 | 0 | 0 |
| **Calcium (mg)** | 273 | 274 | 0 | 0 |

**Supplementary Table 2.** Comparison of characteristics of participants between MG and EG (cross-sectional study 1)

|  | All Participants (*n* = 219) | | Healthy (*n* = 145) | | Obesity (*n* = 37) | | Participants Requiring Support (*n* = 37) | |
| --- | --- | --- | --- | --- | --- | --- | --- | --- |
| **All** | **MG (*n* = 76)** | **EG (*n* = 143)** | **MG (*n* = 54)** | **EG (*n* = 91)** | **MG (*n* = 13)** | **EG (*n* = 24)** | **MG (*n* = 9)** | **EG (*n* = 28)** |
| Age (years) | 73.3 ± 0.7 | 72.1 ± 0.5 | 73.0 ± 0.7 ^#^ | 70.9 ± 0.5 | 71.2 ± 1.8 | 71.0 ± 0.9 | 77.9 ± 3.0 | 77.1 ± 1.5 |
| Hight (cm) | 157.4 ± 1.0 | 156.5 ± 0.6 | 158.2 ± 1.2 | 157.1 ± 0.7 | 157.5 ± 2.1 | 158.7 ± 1.6 | 152.3 ± 2.7 | 152.7 ± 1.7 |
| Body Weight (kg) | 56.8 ± 1.1 | 56.5 ± 0.8 | 54.3 ± 1.1 | 53.1 ± 0.7 | 65.5 ± 1.9 | 67.3 ± 1.7 | 59.6 ± 3.9 | 58.4 ± 1.9 |
| BMI (kg/m^2^) | 22.9 ± 0.4 | 23.0 ± 0.3 | 21.6 ± 0.3 | 21.5 ± 0.2 | 26.4 ± 0.3 | 26.6 ± 0.3 | 25.6 ± 1.6 | 25.1 ± 0.8 |
| %Fat (%) | 29.8 ± 0.9 | 31.3 ± 0.6 | 27.6 ± 1.0 | 29.4 ± 0.6 | 36.5 ± 1.4 | 34.2 ± 1.2 | 33.9 ± 3.0 | 34.9 ± 1.5 |
| **Male** | **MG (*n* = 26)** | **EG (*n* = 43)** | **MG (*n* = 18)** | **EG (*n* = 23)** | **MG (*n* = 4)** | **EG (*n* = 12)** | **MG (*n* = 4)** | **EG (*n* = 8)** |
| Age (years) | 73.8 ± 1.1 | 72.3 ± 0.8 | 73.7 ± 1.3 | 72.3 ± 1.0 | 74.8 ± 2.5 | 70.8 ± 1.3 | 73.5 ± 3.4 | 74.5 ± 2.6 |
| Hight (cm) | 165.7 ± 1.2 | 164.0 ± 0.9 | 166.9 ± 1.4 | 164.2 ± 1.2 | 165.9 ± 1.9 | 164.3 ± 2.0 | 160.4 ± 2.2 | 162.6 ± 1.4 |
| Body Weight (kg) | 62.0 ± 1.7 | 64.1 ± 1.3 | 59.1 ± 1.8 | 60.3 ± 1.2 | 73.8 ± 1.4 | 72.0 ± 2.4 | 63.3 ± 2.6 | 62.9 ± 3.4 |
| BMI (kg/m^2^) | 22.6 ± 0.6 | 23.8 ± 0.4 | 21.2 ± 0.5 | 22.3 ± 0.3 | 26.9 ± 0.8 | 26.5 ± 0.4 | 24.7 ± 1.0 | 23.9 ± 1.3 |
| %Fat (%) | 23.9 ± 1.4 | 26.3 ± 0.8 | 21.2 ± 1.7 | 24.0 ± 1.0 | 31.1 ± 1.3 | 30.1 ± 1.0 | 29.0 ± 2.0 | 27.6 ± 2.4 |
| **Female** | **MG (*n* = 50)** | **EG (*n* = 100)** | **MG (*n* = 36)** | **EG (*n* = 68)** | **MG (*n* = 9)** | **EG (*n* = 12)** | **MG (*n* = 5)** | **EG (*n* = 20)** |
| Age (years) | 73.0 ± 0.9 | 72.0 ± 0.6 | 72.7 ± 0.9 ^#^ | 70.4 ± 0.5 | 69.6 ± 2.2 | 71.1 ± 1.4 | 81.4 ± 4.2 | 78.2 ± 1.9 |
| Hight (cm) | 153.1 ± 0.9 | 153.3 ± 0.6 | 153.9 ± 1.0 | 154.7 ± 0.6 | 153.8 ± 2.0 | 153.1 ± 1.1 | 145.9 ± 1.0 | 148.8 ± 1.5 |
| Body Weight (kg) | 54.1 ± 1.2 | 53.3 ± 0.7 | 51.8 ± 1.1 | 50.7 ± 0.6 | 61.8 ± 1.4 | 62.6 ± 1.5 | 56.6 ± 6.8 | 56.5 ± 2.2 |
| BMI (kg/m^2^) | 23.0 ± 0.4 | 22.7 ± 0.3 | 21.8 ± 0.3 | 21.2 ± 0.2 | 26.1 ± 0.2 | 26.8 ± 0.5 | 26.4 ± 2.9 | 25.5 ± 0.9 |
| %Fat (%) | 32.9 ± 0.9 | 33.4 ± 0.6 | 30.8 ± 0.9 | 31.3 ± 0.6 | 38.8 ± 1.3 | 38.3 ± 1.2 | 37.9 ± 4.7 | 37.8 ± 1.5 |

Values are expressed as the mean ± standard errors. ^#^*P* < 0.05 compared to EG (Mann-Whitney). MG: Morning Group, EG: Evening Group, BMI: Body Mass Index, %Fat: Percentage Fat

**Supplementary Table 3.** Comparison of energy and dietary intake between MG and EG (cross-sectional study 1)

|  | All Participants (*n* = 219) | | Healthy (*n* = 145) | | Obesity (*n* = 37) | | Participants Requiring Support (*n* = 37) | |
| --- | --- | --- | --- | --- | --- | --- | --- | --- |
| **All** | **MG (*n* = 76)** | **EG (*n* = 143)** | **MG (*n* = 54)** | **EG (*n* = 91)** | **MG (*n* = 13)** | **EG (*n* = 24)** | **MG (*n* = 9)** | **EG (*n* = 28)** |
| Energy Intake (kcal/day) | 2048.0 ± 57.7 | 2076.6 ± 41.8 | 2077.6 ± 70.7 | 2102.8 ± 55.4 | 2055 ± 116.2 | 2153.0 ± 92.5 | 1860.7 ± 176.1 | 1925.8 ± 79.9 |
| Carbohydrate intake (g/day) | 261.2 ± 8.1 | 268.2 ± 5.4 | 265.1 ± 9.9 | 265.9 ± 6.8 | 253.8 ± 17.6 | 287.7 ± 13.8 | 249.1 ± 22.8 | 258.9 ± 10.7 |
| Fat Intake (g/day) | 70.6 ± 2.5 | 71.6 ± 2.0 | 71.8 ± 2.9 | 74.6 ± 2.7 | 73.6 ± 5.5 | 68.3 ± 4.3 | 58.4 ± 8.4 | 64.5 ± 3.9 |
| Protein Intake (g/day) | 78.1 ± 2.8 | 74.6 ± 1.8 | 80.3 ± 3.3 | 76.9 ± 2.4 | 74.5 ± 4.9 | 76.1 ± 3.7 | 70.5 ± 10.4 | 66.0 ± 3.1 |
| Breakfast Protein Intake (g/day) | 29.8 ± 1.1 ^###^ | 18.9 ± 0.7 | 30.6 ± 1.3 ^###^ | 18.7 ± 1.0 | 27.4 ± 2.0 ** | 19.6 ± 1.5 | 28.9 ± 4.0 ** | 18.9 ± 1.3 |
| Lunch Protein Intake (g/day) | 26.4 ± 1.4 | 24.9 ± 0.9 | 27.5 ± 1.8 | 26.4 ± 1.1 | 25.4 ± 3.3 | 25.4 ± 2.1 | 21.2 ± 3.8 | 19.6 ± 1.7 |
| Dinner Protein Intake (g/day) | 21.9 ± 1.0 ^###^ | 30.8 ± 0.8 | 22.2 ± 1.2 ^###^ | 31.8 ± 1.0 | 21.6 ± 1.7 ** | 31.0 ± 1.7 | 20.4 ± 3.5 * | 27.5 ± 1.6 |
| Breakfast Protein Intake (g/kg BW/day) | 0.53 ± 0.02 ^###^ | 0.34 ± 0.01 | 0.57 ± 0.02 ^###^ | 0.36 ± 0.02 | 0.42 ± 0.03 ** | 0.30 ± 0.03 | 0.50 ± 0.08 * | 0.34 ± 0.03 |
| Lunch Protein Intake (g/kg BW/day) | 0.48 ± 0.03 | 0.45 ± 0.02 | 0.52 ± 0.04 | 0.50 ± 0.02 | 0.39 ± 0.06 | 0.38 ± 0.03 | 0.38 ± 0.08 | 0.35 ± 0.03 |
| Dinner Protein Intake (g/kg BW/day) | 0.39 ± 0.02 ^###^ | 0.56 ± 0.02 | 0.41 ± 0.02 *** | 0.61 ± 0.02 | 0.33 ± 0.02 ## | 0.47 ± 0.03 | 0.35 ± 0.06 * | 0.49 ± 0.03 |
| **Male** | **MG (*n* = 26)** | **EG (*n* = 43)** | **MG (*n* = 18)** | **EG (*n* = 23)** | **MG (*n* = 4)** | **EG (*n* = 12)** | **MG (*n* = 4)** | **EG (*n* = 8)** |
| Energy Intake (kcal/day) | 2132.3 ± 109.9 | 2119.6 ± 70.7 | 2193.7 ± 141.3 | 2076.3 ± 94.6 | 2290.2 ± 187.8 | 2362.2 ± 129.3 | 1698.2 ± 182.3 | 1880.2 ± 138.9 |
| Carbohydrate intake (g/day) | 271.2 ± 16.1 | 281.9 ± 9.8 | 275.8 ± 22.2 | 269.1 ± 12.4 | 275.5 ± 28.5 | 319.9 ± 20.0 | 246.1 ± 19.0 | 261.7 ± 18.6 |
| Fat Intake (g/day) | 71.1 ± 4.5 | 65.8 ± 3.3 | 74.7 ± 4.7 | 66.6 ± 4.8 | 80.9 ± 12.6 | 70.6 ± 5.9 | 45.6 ± 9.3 | 56.3 ± 6.6 |
| Protein Intake (g/day) | 77.7 ± 4.6 | 73.1 ± 3.1 | 82.9 ± 5.3 | 74.5 ± 4.5 | 78.4 ± 9.5 | 79.4 ± 5.1 | 53.8 ± 9.2 | 59.5 ± 5.0 |
| Breakfast Protein Intake (g/day) | 30.8 ± 1.7 ^###^ | 18.8 ± 1.1 | 32.3 ± 2.0 ^###^ | 18.6 ± 1.5 | 29.7 ± 3.8 | 20.1 ± 2.6 | 25.2 ± 5.5 | 17.5 ± 2.1 |
| Lunch Protein Intake (g/day) | 25.4 ± 2.1 | 24.6 ± 1.8 | 28.0 ± 2.6 | 26.7 ± 2.9 | 25.5 ± 2.8 | 25.5 ± 2.0 | 13.4 ± 2.0 | 17.2 ± 2.8 |
| Dinner Protein Intake (g/day) | 21.6 ± 1.7 ^###^ | 29.7 ± 1.3 | 22.6 ± 2.0 * | 29.2 ± 1.8 | 23.3 ± 3.3 ^#^ | 33.8 ± 2.7 | 15.3 ± 3.7 ^#^ | 24.8 ± 1.0 |
| Breakfast Protein Intake (g/kg BW/day) | 0.50 ± 0.03 *** | 0.30 ± 0.02 | 0.55 ± 0.03 ^###^ | 0.31 ± 0.02 | 0.40 ± 0.05 ^#^ | 0.28 ± 0.04 | 0.41 ± 0.09 | 0.29 ± 0.04 |
| Lunch Protein Intake (g/kg BW/day) | 0.41 ± 0.04 | 0.39 ± 0.03 | 0.47 ± 0.04 | 0.44 ± 0.05 | 0.35 ± 0.04 | 0.35 ± 0.03 | 0.22 ± 0.04 | 0.28 ± 0.04 |
| Dinner Protein Intake (g/kg BW/day) | 0.36 ± 0.03 ^###^ | 0.47 ± 0.02 | 0.39 ± 0.04 | 0.49 ± 0.03 | 0.31 ± 0.04 ^#^ | 0.48 ± 0.05 | 0.25 ± 0.07 ^#^ | 0.40 ± 0.02 |
| **Female** | **MG (*n* = 50)** | **EG (*n* = 100)** | **MG (*n* = 36)** | **EG (*n* = 68)** | **MG (*n* = 9)** | **EG (*n* = 12)** | **MG (*n* = 5)** | **EG (*n* = 20)** |
| Energy Intake (kcal/day) | 2004.2 ± 66.6 | 2058.0 ± 51.6 | 2019.5 ± 78.8 | 2111.7 ± 67.2 | 1950.5 ± 137.7 | 1943.8 ± 105.3 | 1990.8 ± 286.7 | 1944.0 ± 99.0 |
| Carbohydrate intake (g/day) | 256.1 ± 9.0 | 262.3 ± 6.3 | 259.7 ± 10.0 | 264.8 ± 8.1 | 244.2 ± 22.2 | 255.4 ± 14.5 | 251.4 ± 40.5 | 257.7 ± 13.2 |
| Fat Intake (g/day) | 70.3 ± 3.0 | 74.1 ± 2.5 | 70.4 ± 3.7 | 77.3 ± 3.2 | 70.4 ± 6.0 | 66.0 ± 6.3 | 68.7 ± 11.9 | 67.7 ± 4.6 |
| Protein Intake (g/day) | 78.3 ± 3.5 | 75.3 ± 2.2 | 79.0 ± 4.2 | 77.7 ± 2.8 | 72.7 ± 5.9 | 72.8 ± 5.5 | 83.8 ± 15.5 | 68.6 ± 3.8 |
| Breakfast Protein Intake (g/day) | 29.3 ± 1.4 *** | 18.9 ± 0.9 | 29.7 ± 1.6 *** | 18.7 ± 1.2 | 26.4 ± 2.5 * | 19.2 ± 1.8 | 31.8 ± 6.0 ** | 19.4 ± 1.6 |
| Lunch Protein Intake (g/day) | 27.0 ± 1.9 | 25.1 ± 1.0 | 27.3 ± 2.3 | 26.3 ± 1.2 | 25.4 ± 4.7 | 25.4 ± 3.9 | 27.5 ± 5.3 | 20.6 ± 2.1 |
| Dinner Protein Intake (g/day) | 22.0 ± 1.2 *** | 31.3 ± 1.0 | 2.0 ± 1.5 ^###^ | 32.6 ± 1.2 | 20.9 ± 2.0 * | 28.2 ± 1.8 | 24.4 ± 5.1 | 28.5 ± 2.1 |
| Breakfast Protein Intake (g/kg BW/day) | 0.55 ± 0.03 *** | 0.36 ± 0.02 | 0.58 ± 0.03 *** | 0.37 ± 0.02 | 0.43 ± 0.04 * | 0.31 ± 0.03 | 0.58 ± 0.13 * | 0.36 ± 0.03 |
| Lunch Protein Intake (g/kg BW/day) | 0.51 ± 0.04 | 0.48 ± 0.02 | 0.54 ± 0.05 | 0.52 ± 0.02 | 0.42 ± 0.08 | 0.41 ± 0.06 | 0.51 ± 0.12 | 0.37 ± 0.04 |
| Dinner Protein Intake (g/kg BW/day) | 0.41 ± 0.02 *** | 0.60 ± 0.02 | 0.43 ± 0.03 ^###^ | 0.65 ± 0.02 | 0.34 ± 0.03 * | 0.46 ± 0.03 | 0.43 ± 0.08 | 0.52 ± 0.04 |

Values are expressed as the mean ± standard errors. ^*^*P* < 0.05, ^**^*P* < 0.01, ^***^*P* < 0.001 compared to EG (t-test). ^#^*P* < 0.05, ^##^*P* < 0.01, ^###^*P* < 0.001 compared to EG (Mann-Whitney). MG: Morning Group, EG: Evening Group, BW: Body Weight

**Supplementary Table 4.** Comparison of characteristics of participants, energy and dietary intake by number of meals meeting 0.4g/kg BW (Cross-sectional study 2-1)

| **Physical Characteristics** | **0 meal (*n* = 12)** | **1 meal (*n* = 27)** | **2 meal (*n* = 47)** | **3 meal (*n* = 39)** |
| --- | --- | --- | --- | --- |
| Age (years) | 69.6 ± 1.1^#^ | 71.2 ± 0.8 | 69.6 ± 0.7^##^ | 73.1 ± 0.8 |
| Hight (cm) | 154.5 ± 1.5 | 156.5 ± 0.9^$$^ | 154.6 ± 0.7 | 52.2 ± 0.9 |
| Body Weight (kg) | 59.4 ± 2.1 | 54.6 ± 1.3^##^ | 53.5 ± 0.9^&, ##^ | 49.3 ± 0.9^&&&^ |
| BMI (kg/m^2^) | 24.9 ± 1.0 | 22.3 ± 0.5^&^ | 22.4 ± 0.3^&^ | 21.3 ± 0.4^&&^ |
| %Fat (%) | 37.0 ± 1.7 | 32.4 ± 0.9^&^ | 32.7 ± 0.9^&^ | 30.5 ± 0.8^&&&^ |
| Fat Mass (kg) | 22.2 ± 1.7 | 17.9 ± 0.8^†^ | 17.5 ± 0.7^††^ | 15.2 ± 0.5^†††^ |
| **Energy and Dietary Intake** |  |  |  |  |
| Energy Intake (kcal/day) | 1481.1 ± 102.8 | 1835.1 ± 72.1^###^ | 2058.3 ± 60.8^&&&,##^ | 2387.6 ± 78.4^&&&^ |
| Carbohydrate intake (g/day) | 218.3 ± 16.4 | 248.4 ± 11.0^#^ | 251.7 ± 8.6^##^ | 293.9 ± 9.8^&&^ |
| Fat Intake (g/day) | 44.9 ± 3.8 | 62.5 ± 3.6^&&,###^ | 75.1 ± 2.5^&&&,*,##^ | 88.8 ± 4.3^&&&^ |
| Protein Intake (g/day) | 48.1 ± 3.1 | 63.4 ± 2.2^**,###^ | 76.6 ± 2.1^&&&,###^ | 96.5 ± 3.8^&&&^ |

Values are expressed as mean ± standard errors. ^†^*P* < 0.05, ^††^*P* < 0.01, ^†††^*P* < 0.001 compared to 0 meal (One-Way ANOVA), ^$$^*P* < 0.01 compared to 3 meal (One-Way ANOVA), ^&^*P* < 0.05, ^&&^*P* < 0.01, ^&&&^*P* < 0.001 compared to 0 meal (Mann-Whitney), ^*^*P* < 0.05, ^**^*P* < 0.01 compared to 2 meal LD (Mann-Whitney), ^#^*P* < 0.05, ^##^*P* < 0.01, ^###^*P* < 0.001 compared to 3 meal (Mann-Whitney), BMI: Body Mass Index, %Fat: Percentage Fat

**Supplementary Table 5.** Comparison of characteristics of participants, energy and dietary intake between patterns with only one meal of adequate protein intake (cross-sectional study 2-3)

| **Characteristics of participants** | **1 meal B (*n* = 8)** | **1 meal L (*n* = 8)** | **1 meal D (*n* = 11)** |
| --- | --- | --- | --- |
| Age (years) | 71.0 ± 1.1 | 72.5 ± 2.1 | 70.5 ± 1.0 |
| Hight (cm) | 158.5 ± 1.6 | 154.4 ± 1.8 | 156.7 ± 1.3 |
| Body Weight (kg) | 57.8 ± 2.8 | 54.1 ± 2.9 | 52.7 ± 1.5 |
| BMI (kg/m^2^) | 23.0 ± 0.9 | 22.7 ± 1.1 | 21.5 ± 0.7 |
| %Fat (%) | 32.0 ± 1.7 | 33.8 ± 1.6 | 31.7 ± 1.3 |
| Fat Mass (kg) | 18.7 ± 1.6 | 18.5 ± 1.7 | 16.8 ± 1.1 |
| **Nutrient Intake** |  |  |  |
| Energy Intake (kcal/day) | 1702.3 ± 127.3 | 1862.5 ± 114.4 | 1911.8 ± 127.7 |
| Carbohydrate intake (g/day) | 238.4 ± 20.1 | 253.4 ± 18.8 | 252.1 ± 19.3 |
| Fat Intake (g/day) | 55.9 ± 6.9 | 60.5 ± 4.1 | 68.9 ± 6.6 |
| Protein Intake (g/day) | 60.0 ± 3.6 | 66.5 ± 4.4 | 63.7 ± 3.7 |

Values are expressed as the mean ± standard errors. BMI: Body Mass Index, %Fat: Percentage Fat, B: Breakfast, L: Lunch, D: Dinner

**Supplementary Table 6.** Comparison of pre-intervention characteristics of participants, energy and dietary intake (intervention study)

| **Characteristics of participants** | **MPRO (*n* = 10)** | **MPLA (*n* = 11)** | **EPRO (*n* = 9)** | **EPLA (*n* = 10)** |
| --- | --- | --- | --- | --- |
| Age (years) | 69.0 ± 1.1 | 70.2 ± 1.8 | 70.3 ± 1.3 | 68.3 ± 1.0 |
| Hight (cm) | 154.6 ± 1.6 | 155.8 ± 1.3 | 155.5 ± 1.2 | 156.9 ± 1.5 |
| Body Weight (kg) | 55.7 ± 1.9 ^#^ | 57.1 ± 1.7 ^# †^ | 52.4 ± 1.0 | 51.7 ± 1.9 |
| BMI (kg/m^2^) | 23.3 ± 0.8 ^#^ | 23.6 ± 0.8 ^#^ | 21.7 ± 0.5 | 22.0 ± 0.6 |
| %Fat (%) | 36.7 ± 1.3 ^##^ | 33.9 ± 2.1 | 33.6 ± 1.0 | 30.5 ± 1.5 |
| Fat Mass (kg) | 20.5 ± 1.2 ^#^ | 19.1 ± 1.5 | 17.7 ± 0.9 | 15.8 ± 1.2 |
| MEQ (score) | 46.8 ± 2.4 | 47.1 ± 1.5 | 48.3 ± 2.2 | 43.8 ± 2.5 |
| MVPA (min/day) | 80.9 ± 7.1 | 75.3 ± 11.4 | 72.2 ± 7.5 | 62.5 ± 7.0 |
| Step Count (step/day) | 7402.8 ± 774.6 ^#^ | 6425.8 ± 1055.2 | 5817.0 ± 1078.2 | 4348.5 ± 501.9 |
| **Energy and Dietary Intake** |  |  |  |  |
| Energy Intake (kcal/day) | 2004.4 ± 194.3 | 1897.8 ± 97.9 | 1718.1 ± 160.1 | 1766.6 ± 98.9 |
| Carbohydrate intake (g/day) | 253.8 ± 22.9 | 234.1 ± 18.1 | 231.6 ± 20.6 | 222.9 ± 13.3 |
| Fat Intake (g/day) | 73.2 ± 9.2 | 70.6 ± 5.1 | 58.8 ± 8.0 | 64.1 ± 5.4 |
| Protein Intake (g/day) | 75.5 ± 7.0 | 70.9 ± 4.9 | 55.0 ± 5.0 | 66.2 ± 5.1 |
| Breakfast Protein Intake (g/day) | 15.1 ± 1.6 | 15.7 ± 1.5 | 10.5 ± 1.8 | 13.1 ± 2.0 |
| Lunch Protein Intake (g/day) | 30.9 ± 3.9 | 26.7 ± 3.7 | 20.0 ± 2.7 | 24.6 ± 2.8 |
| Dinner Protein Intake (g/day) | 29.4 ± 3.3 | 28.6 ± 2.8 | 24.4 ± 2.4 | 28.5 ± 3.1 |
| Breakfast Protein Intake (g/kg BW/day) | 0.27 ± 0.03 | 0.28 ± 0.03 | 0.20 ± 0.03 | 0.25 ± 0.04 |
| Lunch Protein Intake (g/kg BW/day) | 0.55 ± 0.07 | 0.47 ± 0.07 | 0.38 ± 0.05 | 0.49 ± 0.06 |
| Dinner Protein Intake (g/kg BW/day) | 0.53 ± 0.06 | 0.51 ± 0.06 | 0.47 ± 0.05 | 0.55 ± 0.06 |

Values are expressed as the mean ± standard errors. ^†^*P* < 0.05 compared to EPRO (Mann-Whitney). ^#^*P* < 0.05, ^##^*P* < 0.01 compared to EPLA (Mann-Whitney). MPRO: Morning Protein Intake Group, MPLA: Morning Placebo Intake Group. EPRO: Evening Protein Intake Group, EPLA: Evening Placebo Intake Group, BMI: Body Mass Index, %Fat: Percentage Fat, MEQ: Morningness-Eveningness Questionnaire, MVPA: Moderate to Vigorous Physical Activity, BW: Body Weight

**Supplementary Table 7.** Comparison of pre-intervention muscle mass, muscle strength, and physical functions (intervention study)

| **Physical Functions** | **MPRO (*n* = 10)** | **MPLA (*n* = 11)** | **EPRO (*n* = 9)** | **EPLA (*n* = 10)** |
| --- | --- | --- | --- | --- |
| Muscle Mass (kg) | 18.62 ± 0.66 | 19.95 ± 0.57 | 18.30 ± 0.22 | 19.05 ± 0.69 |
| Muscle Mass (kg/BW) | 0.33 ± 0.01 ^#^ | 0.35 ± 0.01 ^#^ | 0.35 ± 0.01 | 0.37 ± 0.01 |
| SMI (kg/m2) | 7.78 ± 0.22 | 8.22 ± 0.21 | 7.57 ± 0.11 | 7.72 ± 0.18 |
| Appendicular skeletal muscle mass (kg) | 13.66 ± 0.59 | 14.41 ± 0.43 ^†^ | 13.33 ± 0.22 | 14.23 ± 0.60 |
| ASMI (kg/m2) | 5.69 ± 0.17 | 5.93 ± 0.14 | 5.51 ± 0.07 | 5.76 ± 0.17 |
| Hand Grip (kg) | 20.32 ± 0.86 | 18.78 ± 1.15 | 18.52 ± 1.10 | 21.15 ± 1.25 |
| Hand Grip (kg/BW) | 0.37 ± 0.02 | 0.33 ± 0.02^#^ | 0.36 ± 0.02 | 0.41 ± 0.03 |
| Gait Speed (m/s) | 1.42 ± 0.07 | 1.39 ± 0.07 | 1.45 ± 0.05 | 1.34 ± 0.05 |
| Balance Test (sec) | 28.37 ± 10.52 | 29.82 ± 8.39 | 50.07 ± 11.67 | 55.63 ± 13.40 |
| TUG (sec) | 5.83 ± 0.18 | 6.15 ± 0.27 | 5.72 ± 0.25 | 5.73 ± 0.29 |
| The ‘Sit to Stand’ Test (sec) | 10.70 ± 1.03 | 11.97 ± 1.12 | 11.09 ± 0.85 | 10.84 ± 0.86 |

Values are expressed as the mean ± standard errors. ^†^*P* < 0.05 compared to EPRO (Mann-Whitney). ^#^*P* < 0.05, ^##^*P* < 0.01 compared to EPLA (Mann-Whitney). MPRO: Morning Protein Intake Group, MPLA: Morning Placebo Intake Group. EPRO: Evening Protein Intake Group, EPLA: Evening Placebo Intake Group, SMI: Skeletal Muscle Index, ASMI: Appendicular Skeletal Muscle Index, TUG: Time Up and Go

**Supplementary Table 8.** Comparison of blood parameters before and after the intervention (intervention study)

| **Blood Parameters** | **MPRO (*n* = 10)** | | **MPLA (*n* = 11)** | | **EPRO (*n* = 9)** | | **EPLA (*n* = 10)** | |
| --- | --- | --- | --- | --- | --- | --- | --- | --- |
|  | **Pre** | **Post** | **Pre** | **Post** | **Pre** | **Post** | **Pre** | **Post** |
| Insulin (µU/mL) | 5.7 ± 0.9 | 6.5 ± 0.8 | 6.9 ± 1.2 | 7.6 ± 2.0 | 3.7 ± 0.6 | 5.3 ± 0.6 | 4.8 ± 0.7 | 8.3 ± 1.6 |
| glucose (mg/dL) | 96.0 ± 2.3 | 98.0 ± 1.5 | 95.1 ± 2.1 | 97.7 ± 2.7 | 95.2 ± 1.6 | 99.4 ± 1.4 | 96.0 ± 3.8 | 100.7 ± 3.4 |
| HOMA-IR | 1.4 ± 0.2 | 1.6 ± 0.2 | 1.7 ± 0.4 | 2.0 ± 0.6 | 0.9 ± 0.2 | 1.3 ± 0.1 | 1.2 ± 0.2 | 2.1 ± 0.4 |
| GH (ng/mL) | 2.1 ± 0.5 | 1.7 ± 0.5 | 1.3 ± 0.1 | 2.0 ± 0.4 | 1.5 ± 0.3 | 1.5 ± 0.3 | 2.4 ± 1.2 | 1.6 ± 0.3 |

Values are expressed as the mean ± standard errors. MPRO: Morning Protein Intake Group, MPLA: Morning Placebo Intake Group. EPRO: Evening Protein Intake Group, EPLA: Evening Placebo Intake Group, HOMA-IR, GH: Growth Hormone


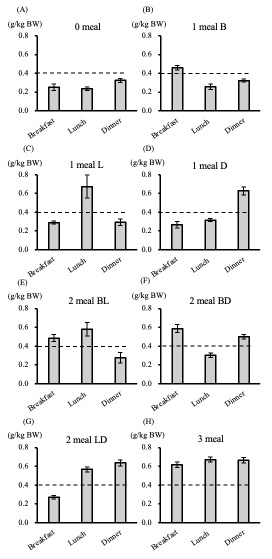


**Supplementary Figure 1.** Protein intake by pattern (cross-sectional study 2-2). (A) 0 meal　(B) 1 meal B　(C) 1 meal L　(D) 1 meal D　(E) 2 meal BL　(F) 2 meal BD　(G) 2 meal LD　(H) 3 meal. Values are expressed as mean and standard errors. BW: Body Weight, B: Breakfast, L: Lunch, D: Dinner, BL: Breakfast & Lunch, BD: Breakfast & Dinner, LD: Lunch & Dinner
